# Characterization of Carbon Nanomaterials Dispersions: Can Metal Decoration of MWCNTs Improve Their Physicochemical Properties?

**DOI:** 10.3390/nano12010099

**Published:** 2021-12-29

**Authors:** Ana T. S. C. Brandão, Sabrina Rosoiu, Renata Costa, A. Fernando Silva, Liana Anicai, Marius Enachescu, Carlos M. Pereira

**Affiliations:** 1 CIQUP—Physical Analytical Chemistry and Electrochemistry Group, Departamento de Química e Bioquimica, Faculdade de Ciências da Universidade do Porto, Rua do Campo Alegre, 687, 4169007 Porto, Portugal; up200706627@edu.fc.up.pt (A.T.S.C.B.); renata.costa@fc.up.pt (R.C.); afssilva@fc.up.pt (A.F.S.); 2 Center for Surface Science and Nanotechnology, University Polytechnica of Bucharest, Splaiul Independentei, 313, 060042 Bucharest, Romania; sabrina.rosoiu@cssnt-upb.ro (S.R.); liana.anicai@cssnt-upb.ro (L.A.); marius.enachescu@cssnt-upb.ro (M.E.); 3 OLV Development SRL, Brasoveni 3, 023613 Bucharest, Romania; 4 Academy of Romanian Scientists, Splaiul Independentei 54, 050094 Bucharest, Romania

**Keywords:** deep eutectic solvent, carbon nanotube, silver nanoparticles, viscosity, ionic conductivity, surface tension, activation energy

## Abstract

A suitable dispersion of carbon materials (e.g., carbon nanotubes (CNTs)) in an appropriate dispersant media, is a prerequisite for many technological applications (e.g., additive purposes, functionalization, mechanical reinforced materials for electrolytes and electrodes for energy storage applications, etc.). Deep eutectic solvents (DES) have been considered as a promising “green” alternative, providing a versatile replacement to volatile organic solvents due to their unique physical-chemical properties, being recognized as low-volatility fluids with great dispersant ability. The present work aims to contribute to appraise the effect of the presence of MWCNTs and Ag-functionalized MWCNTs on the physicochemical properties (viscosity, density, conductivity, surface tension and refractive index) of glyceline (choline chloride and glycerol, 1:2), a Type III DES. To benefit from possible synergetic effects, AgMWCNTs were prepared through pulse reverse electrodeposition of Ag nanoparticles into MWCNTs. Pristine MWCNTs were used as reference material and water as reference dispersant media for comparison purposes. The effect of temperature (20 to 60 °C) and concentration on the physicochemical properties of the carbon dispersions (0.2–1.0 mg cm^−3^) were assessed. In all assessed physicochemical properties, AgMWCNTs outperformed pristine MWCNTs dispersions. A paradoxical effect was found in the viscosity trend in glyceline media, in which a marked decrease in the viscosity was found for the MWCNTs and AgMWCNTs materials at lower temperatures. All physicochemical parameters were statistically analyzed using a two-way analysis of variance (ANOVA), at a 5% level of significance.

## 1. Introduction

Carbon nanotubes (CNTs) were first reported by Iijima [1], receiving much interest due to their properties such as high aspect ratio, lightweight, high tensile strength, excellent electrical conductivity and stability [2,3,4]. The outer layer of the CNTs can be used as an anchor for the deposition of metallic nanoparticles (NPs) [3]. NPs incorporation is of great interest because they can form hybrid nanostructures that exhibit enhanced [5] or novel properties, due to size, large surface area and quantum dimension, which can enhance their physical and chemical properties [6]. Several metals (e.g., Ag, Au, and Pt) have been explored for the decoration of CNTs [7,8,9,10]. However, Ag nanoparticles (Ag-NPs) have received unique attention [11,12,13,14,15,16,17,18] due to their high conductivity, which contributes to the improvement of the electrical conductivity of the CNTs composites.

The electrolyte reaction media considered to entrap metallic NPs into the carbon matrix have also captured great attention due to their high impact on the chemical properties, mechanical stability and compatibility with enhanced surface area and electrical properties [19]. Non-aqueous solvents may present some advantages compared to aqueous solvents [19,20,21], when the dispersibility and the electrochemical stability is taken into consideration [22]. Ionic liquids (ILs) present high production and purification costs, making these dense non-aqueous fluids less competitive when compared with traditional solvents.

Alternatively, deep eutectic solvents (DES) have emerged as suitable and attractive “green” alternatives to ILs for many chemical processes and are often described as ILs analogues in the literature [20]. Due to their unique compositional flexibility, intermolecular interactions and physicochemical properties, DES are pointed as a good dispersion media for carbon materials, such as CNTs [21].

Cojocaru et al. [18] suggested for the first time, the electrochemical synthesis of Ag-NPs involving choline chloride-glycerol based DES using pulse reversed current technique using a two-electrode cell, in which the Ag^+^ ions are produced by anodic dissolution of the Ag metal or dissolution of the Ag-based salt. Poly(*N*-vinyl pyrrolidone) (PVP) was used as a capping agent to avoid agglomeration and to control the growth of the Ag NPs [23]. Following the works by Brandão et al. [24] and Cojocaru et al. [18], the electrochemical decoration of MWCNTs with Ag-NPs from choline chloride–glycerol eutectic mixture, using pulse reversed current in a two-electrode cell can be successfully achieved. Water-glyceline based DES binary systems have been successfully used in assisting and optimizing the dispersion efficiency of carbon nanotubes in water, allowing to predict the fate and transport of SWCNTs in aqueous DES systems [21].

Several studies have been carried out regarding the thermophysical properties of several ILs and DES [25,26,27,28,29,30,31,32,33], and both have been proved to be good dispersant media for CNTs dispersion [21,34,35,36,37,38,39].

CNTs dispersions can be used for many applications, such as lubricants [40,41,42], polymer nanocomposites [43], and more recently it has been drawing the attention of the scientific community for the development of next-generation porous solid-state electrolytes [44,45,46,47] and as electrode materials [48].

To the best of our knowledge, it is the first time that properties such as viscosity, density, conductivity, refractive index, and surface tension of MWCNTs and AgMWCNTs/DES-nanomaterial dispersions in glyceline have been gathered and further compared with water dispersions of the same materials. Further, several methods of AgMWCNTs/DES-nanomaterial synthesis were studied to infer the effect of synthesis parameters on the performance of AgMWCNTs dispersions in DES.

The synthesis of AgMWCNTs in DES is expected to achieve a double goal, as it is predicted to improve the surface wettability of the composite material by the solvent but also to markedly increase the capacitance of interfaces between composite electrodes, containing the metallic nanoparticles decorated carbon nanotubes, and DES [24]. The dispersion of MWCNTs and AgMWCNTs in DES may present a valuable route to obtain dispersions with enhanced properties for application in advanced energy storage devices.

## 2. Materials and Methods

### 2.1. Preparation of DES

Choline chloride (Sigma Aldrich, 99%, Darmstadt, Germany) was dried overnight in the oven, at 60 °C, before use; glycerol (Sigma Aldrich, 99%, Darmstadt, Germany), and poly (N-vinyl pyrrolidone) (PVP 10, Sigma Aldrich, Darmstadt, Germany) were used as received. The eutectic mixture (known as glyceline) was formulated by mixing and heating at 60 °C the ChCl with glycerol, as HBD, in the molar ratio of 1:2, until a homogeneous and clear liquid was formed.

Before the physicochemical studies, glyceline water content (wt.%) was determined using a Karl Fischer titrator (831 KF Coulometer, Methrom (Herisau, Switzerland)). The sample solution was manually mixed to achieve the maximum homogenization before titrating. A 1 cm^3^ of a sample (~1.15 g) was added to the dry methanol solvent (HYDRANAL™, max 0.01 wt.% water, Riedel−de−Haën (Honeywell Specialty Chemicals Seelze GmbH Charlotte, USA)) and titrated with HYDRANAL™ Composite 5 Reagent (4.5–5.5 mg mL^−1^ water equivalent, Riedel−de−Haën (Honeywell Specialty Chemicals Seelze GmbH Charlotte, USA)) for moisture determination. Measurements were performed in triplicate and the water content in glyceline was found to be 6.3 ± 0.5 wt.%.

Commercial MWCNTs (Sigma Aldrich, 99%, Darmstadt, Germany) were dispersed in the glyceline media, followed by ultrasonication for 4 h. The MWCNTs have an average diameter of 24 ± 4 nm, determined by Scanning electron microscopy image analysis (SEM, Hitachi SU 8230 equipment (Krefeld, Germany)).

### 2.2. Electrochemical Synthesis of Ag Nanoparticles and Decoration of MWCNTs

The electrochemical synthesis of Ag-NPs on the surface of MWCNTs was previously described by Brandão et al. [24]. Briefly, AgMWCNTs/DES were synthesized by a pulse reverse current mode (pe 86CB 3HE, plating electronic GmbH), at room temperature, using a two-electrode cell configuration with 50 mL of glyceline. Both electrodes were composed of Ag wires with an exposed area of approximately 5 cm^2^. Before each synthesis, the Ag wires were hand polished and washed with distilled water, and dried.

Different applied anodic and cathodic currents, and different on (t_ON_) and off-times (t_OFF_) were used to prepare the Samples A to E, details of which are presented in Table S1, in Supplementary Materials. All electrodepositions were performed under magnetic stirring or ultrasonication. To recover the AgMWCNT, the electrolyte was centrifuged at 4000 rpm for 20 min, with intermediary washing with ethanol and hot water to remove the eutectic mixture. After AgMWCNT separation, the material was dried overnight at 60 °C and used without any further treatment. The main characteristics (amount, size, and specific capacitance) of the materials A to E are listed in Table 1.

### 2.3. Zeta Potential Analysis

The zeta potential (ZP) of particles was determined by electrophoretic mobility using the Helmholtz–Smoluchowski equation [49] through Zetasizer Nano ZS (Malvern Instruments, Malvern, UK). The values reported are the mean ± standard deviation of three different measurements recorded for each sample.

ZP was not calculated for the carbon-glyceline dispersions, due to the incapability of the equipment to measure these samples.

### 2.4. Physicochemical Characterization

#### 2.4.1. Density and Dynamic Viscosity

Dynamic viscosities (η) and densities (ρ) of MWCNTs and AgMWCNTs dispersed in glyceline, and water were measured using the automated Anton Paar DMA™ 4500 M micro viscometer (Anton Paar GmbH, Graz, Austria). The density measurement is based on electromagnetically induced oscillations of a U-shaped glass tube. The standard deviations associated with the density measurement were below 0.00005 g cm^−3^. The viscosities were measured based on Hoeppler’s falling ball principle. A 100 mm long capillary with a diameter of 3.0 mm (glyceline) or 1.6 mm (water) with a tilting angle from 24° to 70°. The standard deviation in dynamic viscosities data was below 0.5%.

All the physicochemical characterization measurements were performed in the temperature range between 20 °C and 60 °C, with 10 °C steps.

#### 2.4.2. Refractive Index

Refractive index (RI) of both MWCNTs and AgMWCNTs dispersions in glyceline and water were determined using an Abbe Refractometer DR-A1-Plus (Atago Co, Ltd., Tokyo, Japan) with a resolution of ±0.00001 and uncertainty in the experimental measurements of ±0.0002. The apparatus was calibrated by measuring the refractive index of ultrapure water before the measurements, taking into consideration the values of RI vs. temperature of water already published [50].

#### 2.4.3. Surface Tension

The measurement of the surface tension was performed in a homemade analyzer with temperature control, which is composed of a camera (Guppy F-036, Allied-vision, Stadtroda, Germany), and a sample holder. The measurement procedure was calibrated through the measurement of several drops of ultra-pure water at different temperatures. For every dispersion, 5 drops were formed to obtain an average and standard deviation. Once the drop is placed, an image is taken. The image is digitally processed and analyzed using the ImageJ software (W. Rasband, National Institutes of Health, Bethesda, MD, USA) with the Pendent Drop plug-in (Adrian Daerr, Paris, France) [51].

#### 2.4.4. Ionic Conductivity

The ionic conductivity of the MWCNTs dispersions in glyceline and water was measured with Mettler Toledo Conductivity meter F30 (Columbus, OH, USA). An aqueous solution of KCl was used as a calibration standard. The temperature was stabilized with a thermostat for both calibration and experimental measurement.

### 2.5. Statistical Analysis

Statistical analysis (GraphPad Prism 9 software (Graphpad Holdings, San Diego, CA, USA) was employed to perform a two-way analysis of variance (ANOVA) of the experimental data at a 5% level of significance. Linear regression was performed to establish the correlation of every determined parameter with the evolution of temperature and concentration. All determinations were performed in triplicate and a relevant average value and standard deviation are reported.

## 3. Results and Discussion

### 3.1. Morphological Characterization

**Figure 1:**
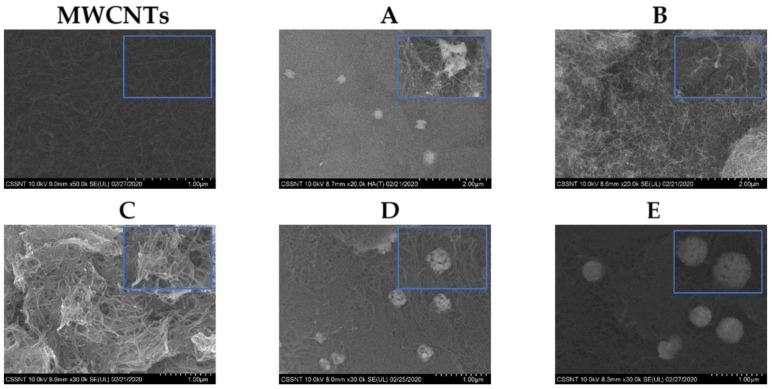
Electron microscopy images showing the structure of MWCNTs and AgMWCNTs samples (**A**–**E**) at different magnifications (20× and 100× (inset)).

The morphological characterization of MWCNTs and Ag-MWCNTs composite samples prepared in this work was performed through SEM analysis, and the images are presented in Figure 1.

The SEM analysis for Samples A to E are displayed in Figure 1, showing the morphology of the Ag-MWCNTs composites with different electrodeposition parameters as detailed in Table 1. SEM analysis of AgMWCNTs (A–E) shows that Ag-NPs are located on the edges and surface of the MWCNTs, and presents some agglomerates of Ag-NPs (more evident in Samples D and E). The average diameter of the Ag-NPs is presented in Table 1. Sample E presents the higher amount of Ag, bigger size of Ag-NPs, and higher specific capacitance as previously published by Brandão et al. [24].

### 3.2. Zeta Potential Analysis

**Figure 2:**
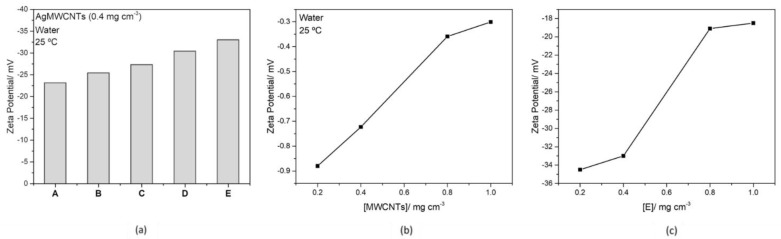
Zeta potential (ZP) for different AgMWCNTs samples (0.4 mg cm^−3^) (**a**), different MWCNTs concentrations (**b**) and different concentrations of Sample E (**c**).

The ZP analysis was performed for MWCNTs and AgMWCNTs samples dispersed in water, and the results are presented in Figure 2. The ZP obtained for the MWCNTs and the different AgMWCNTs samples, using a 0.4 mg cm^−3^ dispersion (Figure 2a) shows that introducing the AgMWCNTs leads to the increase, towards more negative potentials, of ZP. This seems to be strictly associated to the presence of AgNPs, in particular with the increase of Ag NPs size. According to Sabri et al. [52], a high ZP value correlates to stable dispersion and the dispersed MWCNTs are well distributed. While the low ZP derives an attractive force that exceeds the repulsive force, the dispersion of the MWCNTs tends to aggregate. This is in line with the obtained results (Figure 2b,c), where an increase in the concentration of carbon materials in the water dispersions, leads to a decrease in the ZP values.

Figure 2a indicates that the dispersion of Sample E, with a bigger size of Ag NPs, presents a higher value of ZP, and consequently a more stable dispersion. Taking into consideration the overall results, the decoration of MWCNT with Ag nanoparticles significantly changed the zeta potential (from ≈−1 mV to −33 mV AgMWCNT, Sample E). This change in zeta potential for modified MWCNTs is in agreement with other published work [53] and is compatible with increased stability of AgMWCNTs dispersions in water, making it suitable for the characterization of the physicochemical properties.

### 3.3. Physicochemical Properties of MWCNTs and AgMWCNTs

The physicochemical properties of MWCNTs and AgMWCNTs dispersions in glyceline, and water were measured and will be thoroughly discussed in this section. A detailed comparison will be presented for AgMWCNTs Samples A through E since they cover different features of the prepared AgMWCNTs, namely the effect of mass transport on the synthesis step (comparison between Samples B and E); the effect of the amount of Ag and size of Ag-NPs (comparison between Samples C and D, B and E and B and C). The detailed information regarding the parameters for the electrodeposition process of AgMWCNTs can be found in the Supplementary Materials (Table S1).

The detailed information regarding the physicochemical parameters of MWCNTs and AgMWCNTs dispersions can be found in Supplementary Materials, and it will be referred throughout the text as necessary.

#### 3.3.1. Density

Solvent densities (ultrapure water and glyceline) were measured to allow the validation of the experimental procedure and to build a set of reference points to help understand the changes observed.

**Figure 3:**
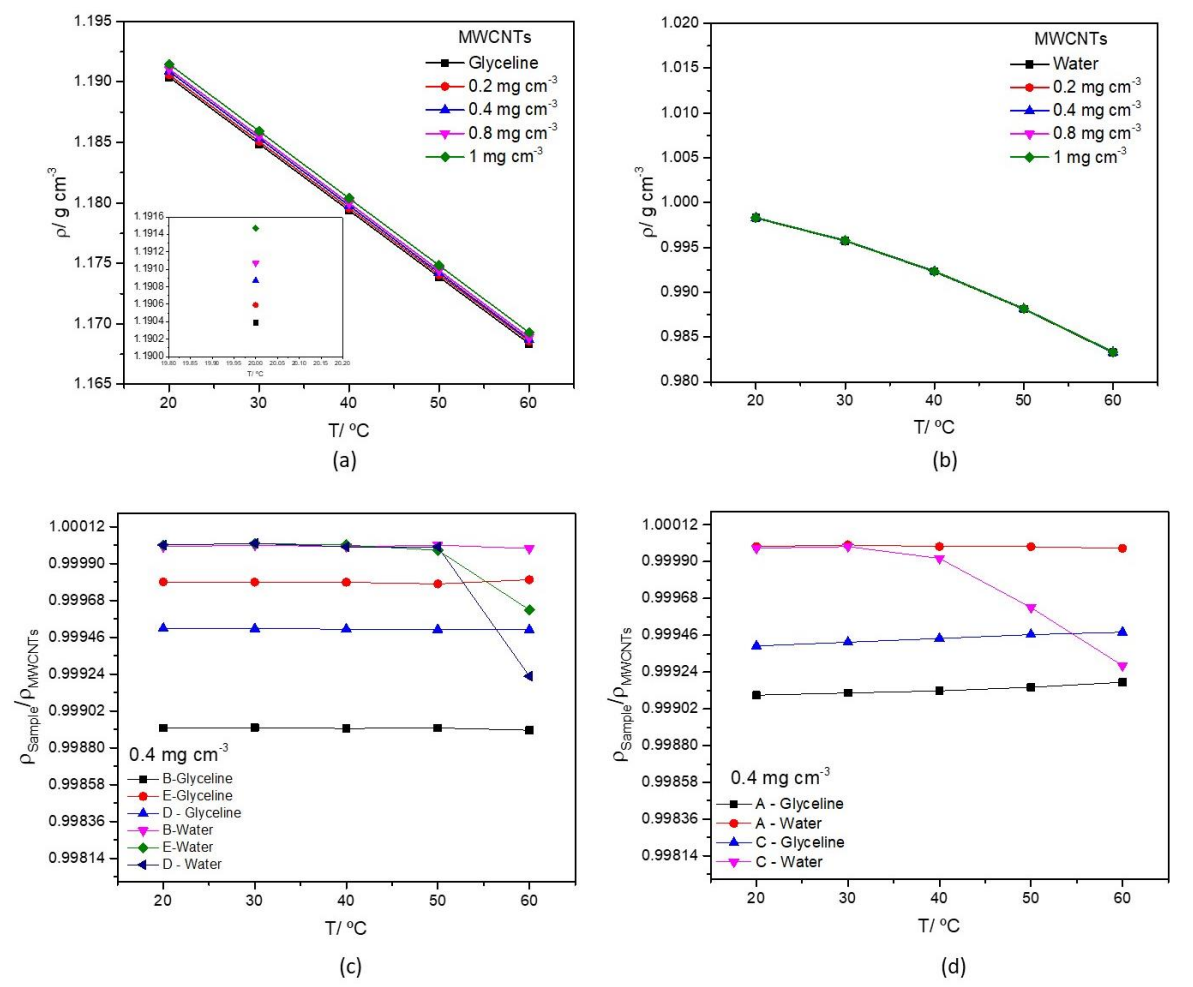
Densities of MWCNTs mixtures (0.2–1.0 mg cm^−3^) in glyceline (**a**), water (**b**) as a function of temperature, and density ratio of AgMWCNTs (0.4 mg cm^−3^) Samples B, D and E (**c**), A and C (**d**) as a function of temperature, in glyceline and water.

The measured density values for MWCNTs dispersed in glyceline and water, as a function of temperature is presented in Figure 3a,b respectively. The temperature dependence in glyceline (Figure 3a) is linear, however, in water (Figure 3b), it presents a slight curve. The linear decrease that can be seen in DES is due to the formation of larger intermolecular voids at higher temperatures, which increase the volume and decrease the density [54]. This effect was already described by several authors [54,55,56,57].

The density values for the different dispersions both in glyceline and water present similar results; however, in both cases, the introduction of the MWCNTs increases the density. The effect of MWCNTs is less pronounced in water, which, as expected, also presents a lower density compared to glyceline. Density values for pure glyceline found in the literature are listed in Table 2 and have a large range of values (namely the values found for 25 °C and 90 °C) and only a reduced number of works (e.g., the work of Crespo et. al. [27] and the present work) display the water content of the eutectic mixture. The value determined in the present work is in good agreement with the value reported by Crespo et. al. [27]. The density of ultrapure water is also in good agreement (less than 0.01% difference) with published values [58].

The decrease in density as a function of temperature is expected since an increase in temperature increases the DES molecules mobility and due to that, the thermal expansion of the DES volume increases [54]. The most important feature of the temperature effect in density is the fact that glyceline and their dispersions have a linear relationship with temperature.

The effect of Ag-NPs attached to the MWCNTs surface, dispersed in glyceline and water media is presented in Figure 3c (Samples B, D and E) and 3d (Samples A and C). To facilitate data interpretation Figure 3c,d present the ratio of ρ_Ag-M WCNTs_/ρ_MWCNTs_ as a function of temperature for samples at a fixed concentration (0.4 mg cm^−3^), a similar effect is observed for all the other concentrations measured under this study. The measured density values of MWCNTs and AgMWCNTs mixtures (Samples A to E) as a function of temperature in the range 20 °C to 60 °C are presented in Supplementary Materials, in Figure S1a–f and Tables S2 and S3 and Figure S2a–f and Tables S4 and S5, respectively for glyceline and water.

Replacing MWCNTs by AgMWCNTs does not introduce any significant effect on the density of water dispersions (less than 0.01%). In glyceline, a slightly larger decrease in density is observed for all the samples in the temperature range between 20 and 60 °C (around 0.0633 ± 0.0003%).

#### 3.3.2. Dynamic Viscosity

Viscosity is an important property that must be addressed, especially for equipment design and fluid flow calculations. Viscosity values for pure glyceline, found in the literature are listed in Table 3. The dynamic viscosity of ultrapure water is also in good agreement (less than 0.05% difference) with published values [58], indicating the quality of the measured values for glyceline and carbon dispersions.

**Figure 4:**
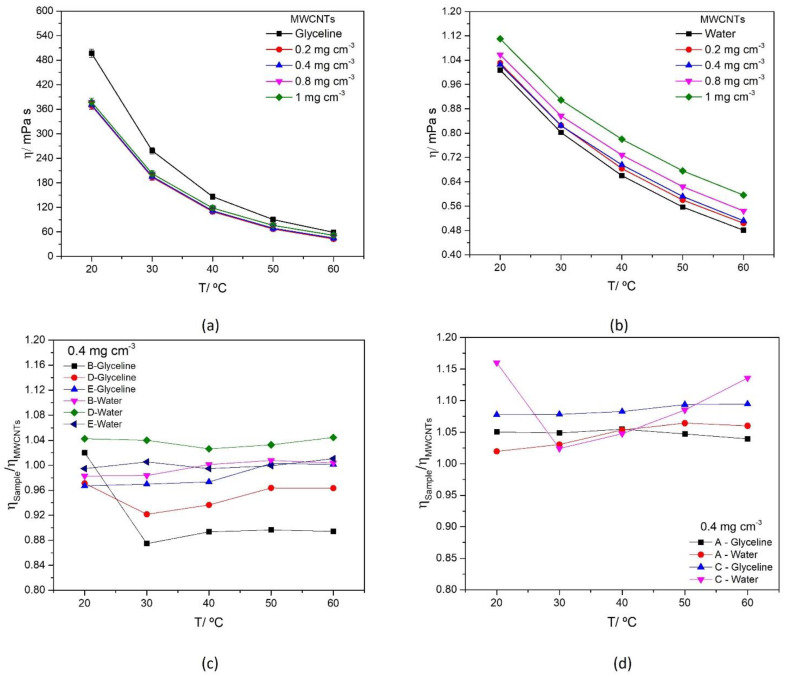
Dynamic viscosity of MWCNTs mixtures (0.2–1.0 mg cm^−3^) in glyceline (**a**), water (**b**) as a function of temperature, and dynamic viscosity ratio of AgMWCNTs (0.4 mg cm^−3^) Samples B, D and E (**c**), A and C (**d**)) as a function of temperature, in glyceline and water.

The viscosity as a function of temperature is presented in Figure 4, for different concentrations of MWCNTs in glyceline and water (Figure 4a,b respectively). The viscosity of water is significantly lower than glyceline, and the decrease of viscosity with the increase in temperature is observed for both media, being more pronounced in glyceline (90% decrease from 20 °C to 60 °C).

The introduction of MWCNTs in the dispersing media displays a different behavior in glyceline and water. There is a decrease in viscosity in glyceline and an increase in water when adding MWCNTs, which is in agreement with data reported in some research papers [61,62]. That inversion observed in glyceline was previously reported by Alizadeh and Moraveji [63], for graphene nanoplatelets dispersed in an ionic liquid which showed a decrease in viscosity when compared to the original ionic liquid. This phenomenon might be explained by the self-lubricating nature of carbonous materials. Lower viscosity can result in reducing the pressure drop penalty in thermo-fluids systems and increasing the hydraulic and energy efficiency as a result [64].

The effect of Ag-NPs attached to the MWCNTs surface, dispersed in glyceline and water media is presented in Figure 4c (Samples B, D and E), 4d (Samples A and C). To facilitate data interpretation Figure 4c,d present the ratio of η_Ag-M WCNTs_/η_MWCNTs_ as a function of temperature for samples at a fixed concentration (0.4 mg cm^−3^). The measured dynamic viscosity values of MWCNTs and AgMWCNTs dispersions (Samples A to E) as a function of temperature in the range 20 °C to 60 °C are presented in SI, in Figure S3f, Tables S6 and S7 for glyceline and Figure S4f, Tables S8 and S9 for water.

The introduction of AgMWCNTs on glyceline media leads to a small increase in viscosity when compared to pristine MWCNTs. AgMWCNTs dispersions in water display a systematic increase in viscosity (when compared to MWCNTs dispersions) of around 1.6%. When this study is carried out in glyceline, the AgMWCNTs samples containing larger amounts of silver (Samples B, D and E) display a decrease in viscosity (a 5.0% decrease) while dispersions prepared with Samples A and C show an increase in viscosity (a 6.7% increase). Samples D and E (which have a larger amount of Ag) display a larger and more consistent decrease in viscosity.

**Figure 5:**
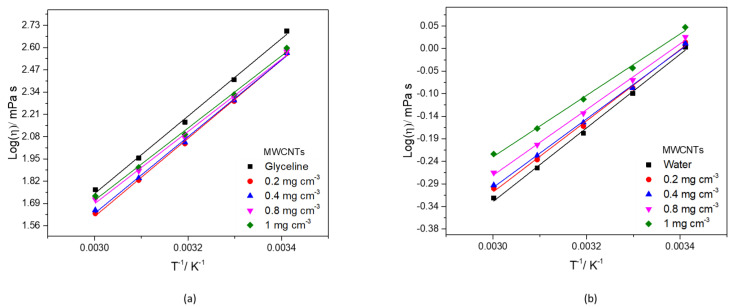
Plot of the logarithm of the viscosities against the reciprocal value of the absolute temperature for glyceline (**a**) and water (**b**) containing MWCNTs with different concentrations.

Studying the viscosity change with temperature allows to retrieve important information that can be retrieved by fitting an Arrhenius type Equation (1):

(1) $$\ln\eta =\ln\eta_{0}+\frac{E_{\eta }}{\mathrm{RT}}$$

where η_0_ is a constant, E_η_ is the energy for activation of the viscous flow, R is the gas constant (8.31 J K^−1^ mol^−1^) and T is the temperature (K) [65]. Figure 5 shows that the data for MWCNTs dispersion in glyceline and water obey Equation (1) well (R^2^ > 0.989).

Table 4 and Table 5 list the values of ln (η_0_) (interception), E_η_/R (slope), and the regression coefficient (R^2^) the model for viscosity/temperature in glyceline and water, respectively. The minimum R^2^ was 0.996, for the higher concentration of MWCNTs in glyceline and water.

The calculated values of E_η_ are summarized in Tables S10 and S11, in Supplementary Materials, for glyceline and water, respectively. The values of E_η_ diminish when the concentration of increases from 0.2 to 1.0 mg cm^−3^. This effect is observed for all samples tested in this study. It is known that lower activation energy is associated with more mobile ions within the melt [66].

Figures S3a–e and S4a–e, in Supplementary Materials, show the fitting data of AgMWCNTs dispersion in glyceline and water, respectively which obey Equation (1) well (R^2^ > 0.989). The values of E_η_ follow the same trend described for the MWCNTs dispersions, diminishing when the concentration increases from 0.2 to 1.0 mg cm^−3^. Tables S12 and S13 list the values of ln (η_0_) (interception), E_η_/R (slope), and the regression coefficient (R^2^) the model for viscosity/temperature in glyceline and water, respectively, for AgMWCNTs dispersions.

#### 3.3.3. Surface Tension

The intermolecular attractive forces in a liquid result in cohesive tension that diminishes the surface area of the liquid’s interface with other phases in contact with the liquid, a phenomenon identified as surface tension, which is measured as the energy required to increase the surface area of a liquid by a unit of area. Surface tension values for pure glyceline and water, found in the literature are listed in Table 6, presenting similar results.

The difference between the experimental values presented for glyceline with the ones reported in the literature may be attributed to a possible difference in the water content of DES however this cannot be verified since other authors did not provide that information.

**Figure 6:**
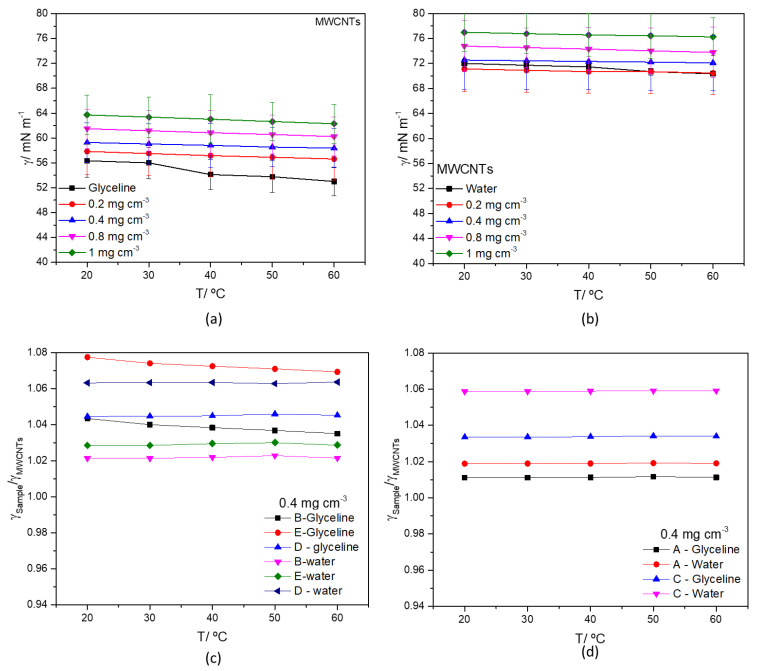
Surface tension of MWCNTs mixtures (0.2–1.0 mg cm^−3^) in glyceline (**a**), water (**b**) as a function of temperature, and surface tension ratio of AgMWCNTs (0.4 mg cm^−3^) Samples B, D and E (**c**) and A and C (**d**) as a function of temperature, in glyceline and water.

Figure 6 shows the effect of concentration and temperature of MWCNTs in glyceline (Figure 6a) and water (Figure 6b). All measured surface tension values of MWCNTs and AgMWCNTs (Samples A to E) as a function of temperature are presented in Supplementary Materials, in Tables S14–S17, respectively for water and glyceline dispersions. Figure S5 presents the surface tension of glyceline/water mixtures with MWCNTs and Ag-MWCNTs as a function of carbon concentration (a) and temperature (b and c).

Figure 6a,b show that both tested solvents display a decrease in surface tension with increasing temperature. The same trend was reported by Abbott et al. [68], where the surface tension is strictly correlated with the viscosity of the system, which will be discussed later. The eight different MWCNTs dispersions (four in each solvent) display the same trend of the pure solvents (Figure 6a,b) showing an increase in surface tension with the increase in MWCNTs concentration. A similar effect was already reported in the literature [69] and is more pronounced in glyceline than in water and reflects the reduced surface activity of the MWCNTs dispersed in glyceline or water. Figure 6c (Samples B, D and E) and 6d (Samples A and C) present the ratio of γ_Ag-M WCNTs_/γ_MWCNTs_ as a function of temperature for AgMWCNTs dispersions at a fixed concentration (0.4 mg cm^−3^). Dispersions of AgMWCNTs display a further increase in surface tension in water (between 2 and 6% increase) and in glyceline (between 1 and 7% increase).

Surface tension data, presented in Figure 6a,b, was analyzed using the “hole theory” developed by Abbott et al. [70,71]. DES “hole theory” was developed by Abbott based on the initial model developed by Fürth [72], which can be used to explain the mobility of ions/particles [70,71]. The “hole theory” model was also considered as a valid model by Bockris et al. [73] to describe high-temperature molten salts. According to this model [74], ionic materials contain empty spaces constituted from thermally generated fluctuations in local density. The vacancies are of random size and its position are in constant motion. It is assumed that an ion can only move through an ionic liquid if it is adjacent to a hole of equal or greater size. The average hole size, r, in a liquid is given by the Equation (2) [71]:

(2) $$4{\pi \langle r}^{2}\rangle =3.5\;\frac{k\;T}{\gamma }$$

where k is the Boltzmann constant, γ is the surface tension, and T is the temperature. The results obtained for glyceline and MWCNTs dispersion are listed in Table 7.

In the studied dispersions, taking into consideration the studied parameters (concentration, temperature, and presence of MWCNTs in glyceline) it is possible to verify, that the void radius decreases with the increase in the concentration of MWCNTs. The results were further compared with pure glyceline. At 20 °C, the DES presents a void radius of 4.216 Å, followed by the MWCNTs dispersion with 4.160 Å, at 0.2 mg cm^−3^. The obtained values of void radius for the dispersion with MWCNTs in glyceline are lower than the carbon-free liquid, indicating that the viscosity decrease is probably controlled by the lubricating properties of MWCNTs [63] rather than the hole size [71].

The values for the average hole size, r, for the AgMWCNTs in glyceline, are presented in Tables S18 and S19 in the Supplementary Materials, revealing that the void radius increases when AgMWCNTs dispersions are used. However, this increase in the void radius is not reflected in a general decrease in viscosity, when compared to MWCNTs dispersions. Samples A and C show an increase in viscosity (Figure 4d) and Samples B, D and E (Figure 4c) show a decrease in viscosity. The presence of AgNPs at MWCNTs surface altered the force balance introducing new surface interactions with the DES components that reduced the MWCNTs lubricating properties but potentiate the increase in the void size. It is the delicate balance between these two opposite effects that define the effect over the dispersion average viscosity.

#### 3.3.4. Ionic Conductivity

Electrochemical applications are an important field where DESs can have a significant contribution to the industry, so there is a great demand for information concerning their electrical and electronic properties, including their ionic conductivity. Due to their relatively high viscosity, DESs have low conductivity. In general, the conductivity of DESs increases as temperature increases [59].

Ionic conductivity values for pure glyceline and water, found in the literature are listed in Table 8. The values for ultrapure water is also in good agreement (less than 1.5% difference) with published values [58]. The ionic conductivity presented by the Milli-Q^®^ water manufacturer is also in good agreement with the obtained result at 20 °C.

The difference between the experimental values presented for glyceline with those reported in the literature may be attributed to a possible difference in the water content of DES this information could not be found and the presence of water in the eutectic mixture can help increase the conductivity [75].

**Figure 7:**
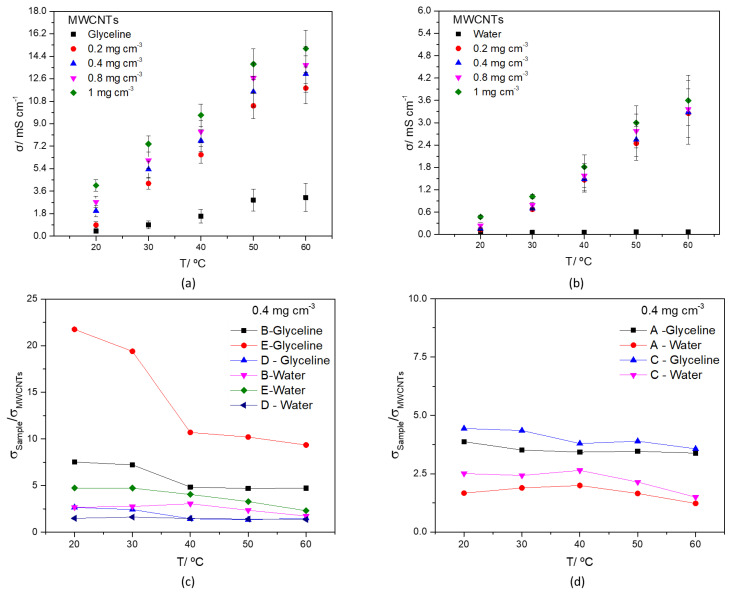
Ionic conductivity of MWCNTs mixtures (0.2–1.0 mg cm^−3^) in glyceline (**a**), water (**b**) as a function of temperature, and ionic conductivity ratio of AgMWCNTs (0.4 mg cm^−3^) Samples B, D and E (**c**) and A and C (**d**) as a function of temperature, in glyceline and water.

The conductivity as a function of temperature is represented in Figure 7, for different concentrations of MWCNTs in glyceline and water (Figure 7a,b respectively). For all the measurements, with increasing temperature, there is an increase in ionic conductivity.

The ionic conductivity of MWCNTs and AgMWCNTs mixtures (samples A to E) as a function of temperature in the range 20 °C to 60 °C are presented in Supplementary Materials, in Tables S20 and S21 and Tables S22 and S23, respectively for water and glyceline.

For all the MWCNTs measurements, in both glyceline and water, with increasing temperature, there is an increase in conductivity. Several studies have presented a considerable increase in conductivity of the fluids with the addition and dispersion of graphene NPs to them [77,78]. The increase of conductivity with the suspension of carbon materials, in this case, MWCNTs, can be attributed to the charge transfer enhancement which is significantly affected by EDL (electrical double layer) effect in solution [79].

The effect of Ag-NPs attached to the MWCNTs surface, dispersed in glyceline and water media is presented in Figure 7c (Samples B, D, and E) and 7d (Samples A, and C). To facilitate data interpretation Figure 7c,d present the ratio of σ_AgMWCNTs_/σ_MWCNTs_ as a function of temperature for samples at a fixed concentration (0.4 mg cm^−3^).

The effect of the presence of the Ag-NPs is very pronounced in this physicochemical property, especially in the glyceline media, showing a decrease in the effect of the Ag-NPs with an increase in temperature. As expected (Figures S6f and S7f, in Supplementary Materials), the Ag-NPs were able to contribute to a substantial increase of the ionic conductivity of dispersion media containing AgMWCNTs due to the high conductivity of silver (r_Ag_ = 6.30 × 10^5^ S cm^−1^) [17].

The ionic conductivity changes, by the introduction of AgMWCNTs, presents an increase up to 20 times, compared to MWCNTs, especially in the sample with a higher amount and size of Ag-NPs (Sample E), followed by the samples with a lower amount of Ag NPs, showing that both parameters are strictly correlated with the ionic conductivity. The increase in conductivity brought by the attachment of Ag-NPs to the MWCNTs surface was also presented earlier [17,80,81,82,83].

**Figure 8:**
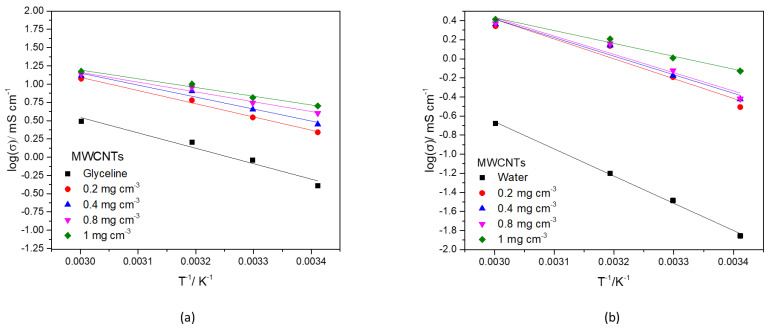
Plot of the logarithm of the conductivity against the reciprocal value of the absolute temperature (1/T) for glyceline (**a**) and water (**b**) containing MWCNTs with different concentrations.

The effect of temperature on the conductivity can be described according to Arrhenius-type behavior (log σ vs. 1/T), as previously developed by Abbott et al. [68,76] and presented in Equation (3).

(3) $$\ln\sigma =\ln\sigma_{0}-\frac{E_{\sigma }}{\mathrm{RT}}$$

where σ is the conductivity at temperature T (K), σ_0_ is the corresponding pre-exponential factor, E_σ_ is the activation energy of ionic conductivity and R is the gas constant (8.31 J K^−1^ mol^−1^). Figure 8a,b show that the data fits Equation (3) accurately (R^2^ > 0.998).

Table 9 and Table 10 lists the values of ln (σ_0_) (interception), E_σ_/R (slope), and the regression coefficient (R^2^) the model for conductivity/temperature in glyceline and water, respectively. The minimum R^2^ was 0.972, for the higher concentrations of MWCNTs in glyceline.

The activation energy of ionic conductivity for the Samples A to E, in glyceline and water, are presented in Table S10 and S11, in Supplementary Materials. The E_σ_ of water is higher, for all MWCNTs concentrations than glyceline. In both cases, there is a decrease in the E_σ_ with an increase in MWCNTs concentration.

Figures S6 and S7 show the fitting data for AgMWCNTs dispersion in glyceline and water, respectively which fits Equation (3) accurately (R^2^ > 0.998). The presence of AgNPs in the MWCNTs significantly improves the Arrhenius-type behavior of glyceline ionic conductivity. Tables S24 and S25 list the values of ln (σ_0_) (interception), E_σ_/R (slope), and the regression coefficient (R^2^) the model for conductivity/temperature in glyceline and water, respectively, for AgMWCNTs dispersions.

**Figure 9:**
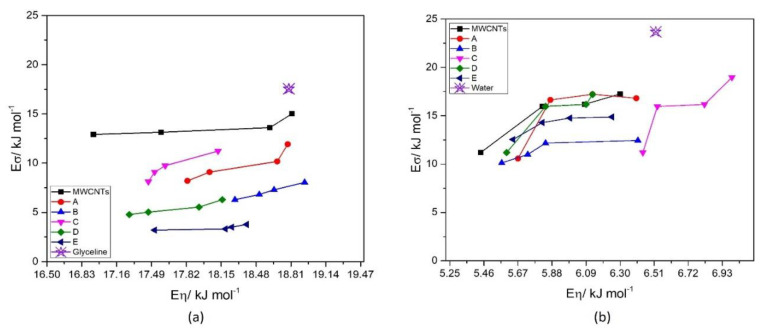
Activation energy of conductivity as a function of activation energy of viscosity for MWCNTs and AgMWCNTs (Samples A to E) in glyceline (**a**) and water (**b**).

Since the conductivity is controlled by the mobility of the charge carriers in ionic fluids, the plot of E_σ_ vs. E_η_ should be linear [68]. That is visible in Figure 9a, for glyceline, containing MWCNTs and AgMWCNTs. However, the same cannot be observed in water, presented in Figure 9b, since it is not an ionic fluid, as presented by Abbott et al. [68].

#### 3.3.5. Refractive Index

**Figure 10:**
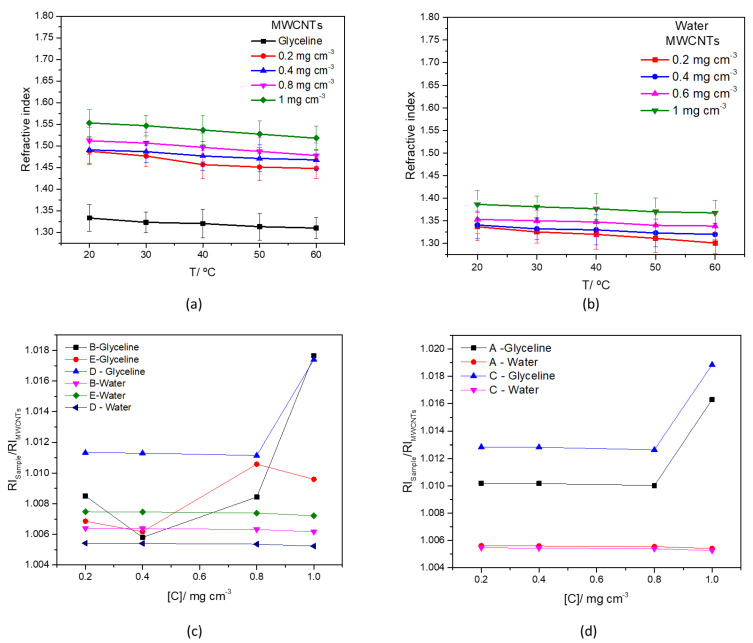
Refractive index of MWCNTs mixtures (0.2–1.0 mg cm^−3^) in glyceline (**a**), water (**b**) as a function of temperature, and refractive index ratio of AgMWCNTs (0.4 mg cm^−3^) Samples B, D and E (**c**) and A and C (**d**) as a function of concentration, in glyceline and water.

The experimental refractive index (RI) data in glyceline and water at room temperature, at different concentrations of MWCNTs, is presented in Figure 10a,b, respectively. Refractive index values for pure glyceline and water, found in the literature are listed in Table 11.

The measured refractive index values of MWCNTs AgMWCNTs mixtures (Samples A to E) as a function of temperature in the range 20 °C to 60 °C, and concentration are presented in Supplementary Materials, in Tables S26–S29, respectively for water and glyceline. Figure S8 presents the effect of concentration of MWCNTs and AgMWCNTs composites on refractive index in glyceline (a) and water (b) media, at 20 °C.

An increase in the concentration of MWCNTs is associated with an increase in the RI, in both glyceline and water. This can be rationalized by the fact that by increasing the concentration of MWCNTs, the availability of carbon materials in solution increases, raising the chances of light to hit a higher number of molecules, and thereby increasing the RI [81]. The effect of temperature was performed for MWCNTs in both glyceline and water, showing that increasing the temperature, the molecules speed increases in solution, causing the light to hit fewer molecules, and thereby reducing the RI [82,83].

The increasing trend of RI with increasing concentration of MWCNTs is also found in the literature [67]. The experimental RI data in glyceline and water at room temperature, at different concentrations of MWCNTs and AgMWCNTs, is presented in Figure S8. It can be observed that the increase in the concentration of MWCNTs and AgMWCNTs is associated with an increase in the RI, in both glyceline and water.

The effect of Ag-NPs attached to the MWCNTs surface, dispersed in glyceline and water media can be observed by analysis of Figure 10c (Samples B, D and E), 10d (Samples A and C). To facilitate data interpretation, Figure 10c,d present the ratio of RI_AgM WCNTs_/RI_MWCNTs_ as a function of temperature for samples at a fixed concentration (0.4 mg cm^−3^).

Shahriari et al. [85] concluded that, with the growth of Ag-NPs size, there is an increase of the RI, due to the increase of diffusivity of the medium. That correlation can be observed for the AgMWCNTs samples in glyceline, especially for Samples B, C and D; however, it was expected that behavior for Sample E, which presents the bigger size of AgNPs, and that is not the case.

### 3.4. Statistical Analysis

The analysis of variance through two-way ANOVA, for viscosity, density, conductivity, and surface tension, as a function of the concentration of MWCNTs and AgMWCNTs (Samples A to E), are shown in Tables S30 and S31, in Supplementary Materials, for glyceline and water, respectively, with 5% level of significance. The statistical significance of the different studied physicochemical parameters depends on the *p*-value.

Starting with the analysis of the results from glyceline media, the concentration of both MWCNTs and AgMWCNTs is statistically significant in defining the properties of viscosity, conductivity, and surface tension, while the effect of temperature is statistically significant in all the studied parameters, presenting a *p*-value < 0.05. The interaction between concentration and temperature is statistically significant only in conductivity and surface tension.

The same conclusions cannot be taken when referring to water media. The effect of concentration and temperature on viscosity, density and conductivity are not statistically significant (*p*-value > 0.05), as proved by the experimental data, meaning that other experimental factors that were not considered in this study are affecting the physicochemical properties under evaluation. The only parameter in which both factors are statistically significant, including its interaction, is the surface tension, with a *p*-value < 0.0001.

For both dispersion media, the statistical analysis corroborates the experimental data obtained in this work.

## 4. Conclusions

The effect of MWCNTs and AgMWCNTs composites on the physicochemical properties of choline chloride-based DES, glyceline, and water was investigated, in the range of concentration from 0.2 to 1.0 mg cm^−3^. The experimental results could be interpreted in terms of the hole theory, in which the ion transfer in DES is limited by the availability of holes of suitable size within the structure of the liquid, nevertheless, the effect of MWCNTs on viscosity and conductivity cannot be explained solely in terms of the hole theory and the contribution of the self-lubricating nature of carbonous materials has to be considered to explain the experimental results.

The measurements of viscosity, density, conductivity, surface tension and refractive index have been conducted. An increase in temperature was shown to result in a decrease in viscosity, density, and surface tension. The conductivity increases with an increase in temperature since it is controlled by ionic mobility.

The introduction of MWCNTs dispersions to glyceline and water led to an increase in conductivity, surface tension and refraction index. However, in glyceline, the presence of carbon nanomaterials led to a decrease in viscosity. The same study carried out in water led to an increase in viscosity, proving the exceptional properties of this choline chloride-based DES.

In general, the use of AgMWCNTs leads to a slight improvement of the dispersion properties, when compared to the use of MWCNTs dispersions. Conductivity is the physicochemical property that benefits the most from the use of AgMWCNTs, allowing to achieve up to 20 × enhancement of conductivity when MWCNTs dispersions are replaced by AgMWCNTs dispersions.

The dispersion of MWCNTs and AgMWCNTs is an exciting area of future research, in particular, their dispersion in eutectic mixtures. The obtained physicochemical properties of the MWCNTs/AgMWCNTs-glyceline dispersions will path the way to achieve exciting breakthroughs in carbon dispersions in the coming years, for several applications, especially in solid-state electrolytes and electrodes for energy storage devices.

## Figures and Tables

**Table 1:** Characteristics of the Ag NPs, particularly Ag NPs content, size, and specific capacitance of the different samples. Data from Brandão et al. [24] *.

Samples	Ag NPs Amount/wt. % (from EDX Analysis)	Ag NPs Size/nm (from SEM Image)	Specific Capacitance /F g^−1^ (using 3 Electrode Cell)
A	3 ± 1	12 ± 5	5.2 ± 0.3
B	11 ± 3	16 ± 4	14.6 ± 1.0
C	1.0 ± 0.2	18 ± 3	7.1 ± 0.8
D	28 ± 8	33 ± 6	16.3 ± 1.2
E	24 ± 9	46 ± 7	28.5 ± 1.6

* Reprinted from Characterization and electrochemical studies of MWCNTs decorated with Ag nanoparticles through pulse reversed current electrodeposition using a deep eutectic solvent for energy storage applications, Pages No. 342–359, Copyright (2021), with permission from Elsevier.

**Table 2:** Density values for glyceline and water at different temperatures (published and obtained results).

Solvent	ρ/g cm^−3^	T/K	Reference	Solvent	ρ/g cm^−3^	T/K	Reference
glyceline	1.198	293	[27]	water	0.99829	293	This work
	1.190		This work		0.99821		[58]
					
	1.180	298	[59]		0.99232	313	This work
	1.190		[60]		0.99222		[58]
					
	1.130	363	[60]		0.98329	333	This work
	1.190		[27]		0.98320		[58]

**Table 3:** Dynamic viscosity values for glyceline and water at different temperatures (published and obtained results).

Solvent	η/mPa s	T/K	Reference	Solvent	η/mPa s	T/K	Reference
glyceline	497	293	This work	Water	1.0016	293	[58]
					1.0074		This work
	325	298	[59]				
	423		[60]		0.7972	303	[58]
					0.8023		This work
	259	303	This work				
					0.6527	313	[58]
	146	313	This work		0.6604		This work

**Table 4:** Viscosity–temperature model parameters for MWCNTs dispersed in glyceline.

	Glyceline	MWCNTs Concentration/mg cm^−3^			
		0.2	0.4	0.8	1.0
ln (η_0_)	−5.0 ± 0.2	−5.2 ± 0.2	−5.1 ± 0.2	−4.6 ± 0.2	−4.6 ± 0.2
(E_η_/R)/K	2261 ± 63	2281 ± 48	2239 ± 54	2109 ± 72	2119 ± 75
R^2^	0.997	0.998	0.998	0.996	0.996

**Table 5:** Viscosity–temperature model parameters for MWCNTs dispersed in water.

	Water	MWCNTs Concentration/mg cm^−3^			
		0.2	0.4	0.8	1.0
ln (η_0_)	−2.68 ± 0.07	−2.58 ± 0.07	−2.49 ± 0.06	−2.37 ± 0.06	−2.19 ±0.06
(E_η_/R)/K	784 ± 21	758 ± 22	732 ± 18	700 ± 19	654 ± 20
R^2^	0.997	0.997	0.998	0.997	0.996

**Table 6:** Surface tension values for glyceline and water at different temperatures (published and obtained results).

Solvent	γ/mN m^−1^	T/K	Reference	Solvent	γ/mN m^−1^	T/K	Reference
glyceline	57.80	293	[67]	water		-	-
	56.33		This work		-		
			
	68.10	298	[59]		71.19	303	[58]
	59.01		[60]		71.54		This work
					
	54.11	313	This work		69.60	313	[58]
					69.88		This work
					
	53.77	323	This work		67.94	323	[58]
					68.19		This work
					
	53.31	333	This work		64.48	333	[58]
					65.43		This work

**Table 7:** Average hole size (Å) according to the hole theory for pure glyceline and several dispersions prepared with increasing concentration of MWCNTs at different temperatures.

	Glyceline	MWCNTs Concentration/mg cm^−3^			
T/°C		0.2	0.4	0.8	1.0
20	4.216	4.160	4.110	4.035	3.964
30	4.300	4.244	4.188	4.114	4.042
40	4.446	4.326	4.265	4.193	4.119
50	4.531	4.405	4.342	4.269	4.197
60	4.634	4.483	4.415	4.347	4.274

**Table 8:** Ionic conductivity values for glyceline and water at different temperatures (published and obtained results).

Solvent	σ/mS cm^−1^	T/K	Reference	Solvent	σ/µS cm^−1^	T/K	Reference
glyceline	0.413	293	This work	water	0.045	293	[58]
					0.050		This work
	2.039	298	[59]		0.056		Milli-Q^®^ water *
	0.981		[60]				
	0.850		[76]		0.061	303	[58]
					0.055		This work
	0.433	303	This work				
					0.070	313	[58]
	1.501	313	This work		0.061		This work
					
	1.914	323	This work		0.075	323	[58]
					0.067		This work
	3.099	333	This work				
	3.441		[76]		0.083	333	[58]
					0.071		This work
	7.652	343	[59]				
	4.520		[60]				

* https://www.merckmillipore.com/PT/en/product/Milli-Q-Reference-Water-Purification-System,MM_NF-Z00QSV0WW. (Last accessed at 10 November 2021).

**Table 9:** Conductivity–temperature model parameters for MWCNTs dispersed in glyceline.

		MWCNTs Concentration/mg cm^−3^			
	Glyceline	0.2	0.4	0.8	1.0
ln (σ_0_)	6.9 ± 0.9	6.5 ± 0.3	6.1 ± 0.6	5.2 ± 0.5	4.8 ± 0.4
(E_σ_/R)/K	−2111 ± 274	−1806 ± 85	−1638 ±193	−1340 ± 144	−1194 ± 110
R^2^	0.989	0.991	0.979	0.977	0.972

**Table 10:** Conductivity–temperature model parameters for MWCNTs dispersed in water.

		MWCNTs Concentration/mg cm^−3^			
	Water	0.2	0.4	0.8	1.0
ln (σ_0_)	7.9 ± 0.3	7 ± 1	6.3 ± 0.9	6 ± 1	4.5 ± 0.3
(E_σ_/R)/K	−2848 ± 95	−2076 ± 366	−1945 ± 286	−1921 ± 308	−1347 ± 104
R^2^	0.997	0.981	0.979	0.982	0.991

**Table 11:** Refractive index values for glyceline and water at different temperatures (published and obtained results).

Solvent	RI	T/K	Reference	Solvent	RI	T/K	Reference
glyceline	1.3331	293	This work	water	1.3321	293	[58]
	1.3325	298	[84]		1.3310	298	
				
	1.3231	303	This work		1.3242	303	
	1.3319		[84]				
				
	1.3201	313	This work		1.3229	313	
	
	1.3305		[84]				
				
	1.3131	323	This work		1.3214	323	
	1.3289		[84]				
				
	1.3099	333	This work		1.3197	333	
	1.3269		[84]				

## Data Availability

Not applicable.

## Supplementary Materials

The following supporting information can be downloaded at: https://www.mdpi.com/article/10.3390/nano12010099/s1, Table S1: parameters for the electrodeposition process of AgMWCNTs. Data from Brandão et al. [24] ** Figure S1: densities (ρ/g cm^−3^) of A–E mixtures (a–e) and comparison between samples at 0.4 mg cm^−3^ (f) in glyceline as a function of temperature; Table S2: densities of MWCNTs and AgMWCNTs mixtures in glyceline as a function of temperature for 0.4 mg cm^−3^; Table S3: densities of MWCNTs mixtures (0.2–1.0 mg cm^−3^) in glyceline as a function of temperature; Figure S2: densities (ρ/g cm^−3^) of A–E mixtures (a–e) and comparison between samples at 0.4 mg cm^−3^ (f) in water as a function of temperature; Table S4: densities of MWCNTs and AgMWCNTs mixtures in water as a function of temperature for 0.4 mg cm^−3^; Table S5: densities of MWCNTs mixtures (0.2–1.0 mg cm^−3^) in water as a function of temperature; Figure S3: plot of logarithm of the viscosity against the reciprocal value of the absolute temperature (1/T) for of A–E mixtures (a–e) and comparison between samples at 0.4 mg cm^−3^ (f) in glyceline; Table S6: viscosity of MWCNTs and AgMWCNTs mixtures in glyceline as a function of temperature for 0.4 mg cm^−3^; Table S7: viscosity of MWCNTs mixtures (0.2–1.0 mg cm^−3^) in glyceline as a function of temperature; Figure S4: plot of logarithm of the viscosity against the reciprocal value of the absolute temperature (1/T) for of A–E mixtures (a–e) and comparison between samples at 0.4 mg cm^−3^ (f) in water; Table S8: viscosity of MWCNTs and AgMWCNTs mixtures in water as a function of temperature for 0.4 mg cm^−3^; Table S9: viscosity of MWCNTs mixtures (0.2–1.0 mg cm^−3^) in water as a function of temperature; Table S10: the calculated values of the activation energies of viscous flow (E_η_) and conductivity (Eτ) at different concentrations of MWCNTs and AgMWCNTs composites (A–E), in glyceline; Table S11: the calculated values of the activation energies of viscous flow (E_η_) and conductivity (Eτ) at different concentrations of MWCNTs and AgMWCNTs composites (A–E), in water.; Table S12: viscosity–temperature model parameters for AgMWCNTs samples dispersed in glyceline; Table S13: viscosity–temperature model parameters for AgMWCNTs samples dispersed in water; Table S14: surface tension of MWCNTs mixtures (0.2–1.0 mg cm^−3^) in water as a function of temperature; Table S15: surface tension of MWCNTs and AgMWCNTs mixtures in water as a function of temperature for 0.4 mg cm^−3^; Table S16: surface tension of MWCNTs mixtures (0.2–1.0 mg cm^−3^) in glyceline as a function of temperature; Table S17: surface tension of MWCNTs and AgMWCNTs mixtures in glyceline as a function of temperature for 0.4 mg cm^−3^; Figure S5: surface tension of glyceline/water mixtures with MWCNTs and AgMWCNTs as a function of carbon concentration (a) and temperature (b and c); Table S18: average hole size (Å) according to the hole theory for glyceline at different concentrations of AgMWCNTs composites (A–E) at 20 °C; Table S19: average hole size (Å) according to the hole theory for glyceline at different temperatures (20–60 °C) of AgMWCNTs composites (A–E) at 0.4 mg cm^−3^; Table S20: conductivity of MWCNTs mixtures (0.2–1.0 mg cm^−3^) in water as a function of temperature; Table S21: conductivity of MWCNTs and AgMWCNTs mixtures in water as a function of temperature for 0.4 mg cm^−3^; Table S22: conductivity of MWCNTs mixtures (0.2–1.0 mg cm^−3^) in glyceline as a function of temperature; Table S23: conductivity of MWCNTs and AgMWCNTs mixtures in glyceline as a function of temperature for 0.4 mg cm^−3^; Figure S6: plot of logarithm of the conductivity against the reciprocal value of the absolute temperature (1/T) for of A–E mixtures (a–e) and comparison between samples at 0.4 mg cm^−3^ (f) in glyceline; Figure S7: plot of logarithm of the conductivity against the reciprocal value of the absolute temperature (1/T) for of A–E mixtures (a–e) and comparison between samples at 0.4 mg cm^−3^ (f) in water; Table S24: conductivity–temperature model parameters for AgMWCNTs samples dispersed in glyceline; Table S25: conductivity–temperature model parameters for AgMWCNTs samples dispersed in water; Table S26: refractive index of MWCNTs mixtures (0.2–1.0 mg cm^−3^) in water as a function of temperature; Table S27: refractive index of MWCNTs and AgMWCNTs mixtures in water as a function of concentration at 20 °C; Table S28: refractive index of MWCNTs mixtures (0.2–1.0 mg cm^−3^) in glyceline as a function of temperature; Table S29: refractive index of MWCNTs and AgMWCNTs mixtures in glyceline as a function of concentration at 20 °C; Figure S8: effect of concentration of MWCNTs and AgMWCNTs composites on refractive index in glyceline (a) and water (b) media, at 20 °C; Table S30: two-way ANOVA between the groups of MWCNTs and AgMWCNTs samples dispersed in glyceline with different temperatures (20–60 °C) and concentrations (0–1.0 mg cm^−3^); Table S31: two-way ANOVA between the groups of MWCNTs and AgMWCNTs samples dispersed in water with different temperatures (20–60 °C) and concentrations (0–1.0 mg cm^−3^).
