# Supplementary material for: Characterization of Carbon Nanomaterials Dispersions: Can Metal Decoration of MWCNTs Improve Their Physicochemical Properties?
Source: Nanomaterials (Basel). 2021 Dec 29;12(1):99. doi: 10.3390/nano12010099 (PMC8746781; doi:10.3390/nano12010099)
Supplement: Supplementary file 1 [file nanomaterials-12-00099-s001.zip › nanomaterials-1484471-supplementary.pdf]

Supplemental Materials

Characterization of Carbon Nanomaterials Dispersions: Can Metal Decoration of MWCNTs Improve Their Physicochemical Properties?

Ana T. S. C. Brandão [1], Sabrina Rosoiu [2], Renata Costa [1], A. Fernando Silva [1], Liana Anicai [2,3], Marius Enachescu [2,4] and Carlos M. Pereira [1,*]

[1] CIQUP–Physical Analytical Chemistry and Electrochemistry Group, Departamento de Química e Bioquimica, Faculdade de Ciências da Universidade do Porto, Rua do Campo Alegre, 687, 4169007 Porto, Portugal; up200706627@edu.fc.up.pt (A.T.S.C.B.); renata.costa@fc.up.pt (R.C.); afssilva@fc.up.pt (A.F.S.)
[2] Center for Surface Science and Nanotechnology, University Polytechnica of Bucharest, Splaiul Independentei, 313, 060042 Bucharest, Romania; sabrina.rosoiu@cssnt-upb.ro (S.R.); liana.anicai@cssnt-upb.ro (L.A.); marius.enachescu@cssnt-upb.ro (M.E.)
[3] OLV Development SRL, Brasoveni 3, 023613 Bucharest, Romania
[4] Academy of Romanian Scientists, Splaiul Independentei 54, 050094 Bucharest, Romania
* Correspondence: cmpereir@fc.up.pt

Electrodeposition parameters 2
Physicochemical Properties 3
Statistical analysis 28

Electrodeposition parameters

Table S1. Parameters for the electrodeposition process of AgMWCNTs. Data from Brandão et al. [1] **.

**Sample**	**t_{ON} (anodic and cathodic) / ms**	**t_{OFF} / ms**	**Current (anodic and cathodic) (i_{ON}) / mA**	**Overall time / min**
A	100		±100	30
B	100		±100	60
C	100	200	±200	60
D	200		±100	60
E*	100		±100	60

* ultrasounds were used during electrodeposition.

** Reprinted from Characterization and electrochemical studies of MWCNTs decorated with Ag nanoparticles through pulse reversed current electrodeposition using a deep eutectic solvent for energy storage applications, Pages No. 342 - 359, Copyright (2021), with permission from Elsevier

1. Brandão, A.T.S.C.; Rosoiu, S.; Costa, R.; Lazar, O.A.; Silva, A.F.; Anicai, L.; Pereira, C.M.; Enachescu, M. Characterization and electrochemical studies of MWCNTs decorated with Ag nanoparticles through pulse reversed current electrodeposition using a deep eutectic solvent for energy storage applications. *J. Mater. Res. Technol.* **2021**.

Physicochemical Properties

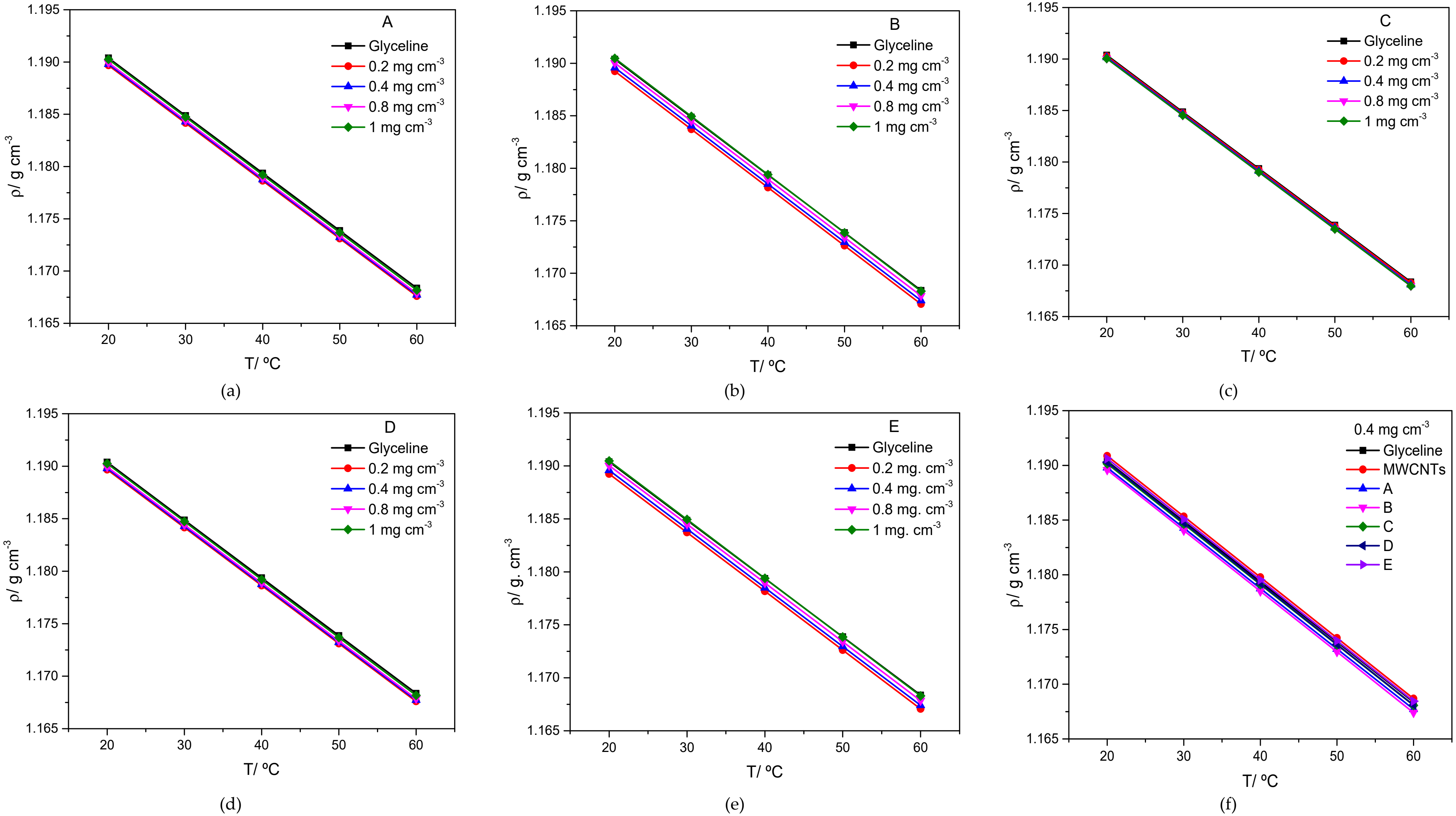

Figure S1. Densities (ρ/g cm^{-3}) of A – E mixtures (a-e) and comparison between samples at 0.4 mg cm^{-3} (f) in glyceline as a function of temperature.

Table S2. Densities of MWCNTs and AgMWCNTs mixtures in glyceline as a function of temperature for 0.4 mg cm^{-3}

	**Glyceline**	**MWCNTs**	**A**	**B**	**C**	**D**	**E**
**Temperature/ °C**				**Density / g cm^{-3}**			
**60**	1.1684 ± 0.0098	1.1687 ± 0.0094	1.1677 ± 0.0104	1.1674 ± 0.0020	1.1681 ± 0.0090	1.1681 ± 0.0098	1.1685 ± 0.0045
**50**	1.1739 ± 0.0077	1.1742 ± 0.0079	1.1732 ± 0.0111	1.1730 ± 0.0109	1.1736± 0.0131	1.1737 ± 0.0089	1.1740 ± 0.0094
**40**	1.1794 ± 0.0089	1.1798 ± 0.0090	1.1788 ± 0.0078	1.1785 ± 0.0045	1.1791 ± 0.0079	1.1792 ± 0.0111	1.1795 ± 0.0079
**30**	1.1841 ± 0.0097	1.1853 ± 0.0085	1.1843 ± 0.0059	1.1841 ± 0.0069	1.1846 ± 0.0091	1.1848 ± 0.0243	1.1851 ± 0.0057
**20**	1.1904 ± 0.0067	1.1909 ± 0.0098	1.1898 ± 0.0088	1.1896 ± 0.0112	1.1902 ± 0.0015	1.1903 ± 0.0156	1.1906 ± 0.0045

Table S3. Densities of MWCNTs mixtures (0.2 – 1.0 mg cm^{-3}) in glyceline as a function of temperature

	**Glyceline**	**0.2 mg cm^{-3}**	**0.4 mg cm^{-3}**	**0.8 mg cm^{-3}**	**1.0 mg cm^{-3}**
**Temperature/ °C**			**Density / g cm^{-3}**		
**60**	1.1684 ± 0.0098	1.1686 ± 0.0058	1.1687 ± 0.0094	1.1689 ± 0.0122	1.1693 ± 0.0090
**50**	1.1739 ± 0.0077	1.1741 ± 0.0231	1.1742 ± 0.0079	1.1744 ± 0.0143	1.1748 ± 0.0079
**40**	1.1794 ± 0.0089	1.1796 ± 0.0096	1.1798 ± 0.0090	1.1800 ± 0.0121	1.1804 ± 0.0046
**30**	1.1841 ± 0.0097	1.1851 ± 0.0045	1.1853 ± 0.0085	1.1855 ± 0.0057	1.1859 ± 0.0022
**20**	1.1904 ± 0.0067	1.1906 ± 0.0034	1.1909 ± 0.0098	1.1911 ± 0.0044	1.1915 ± 0.0079

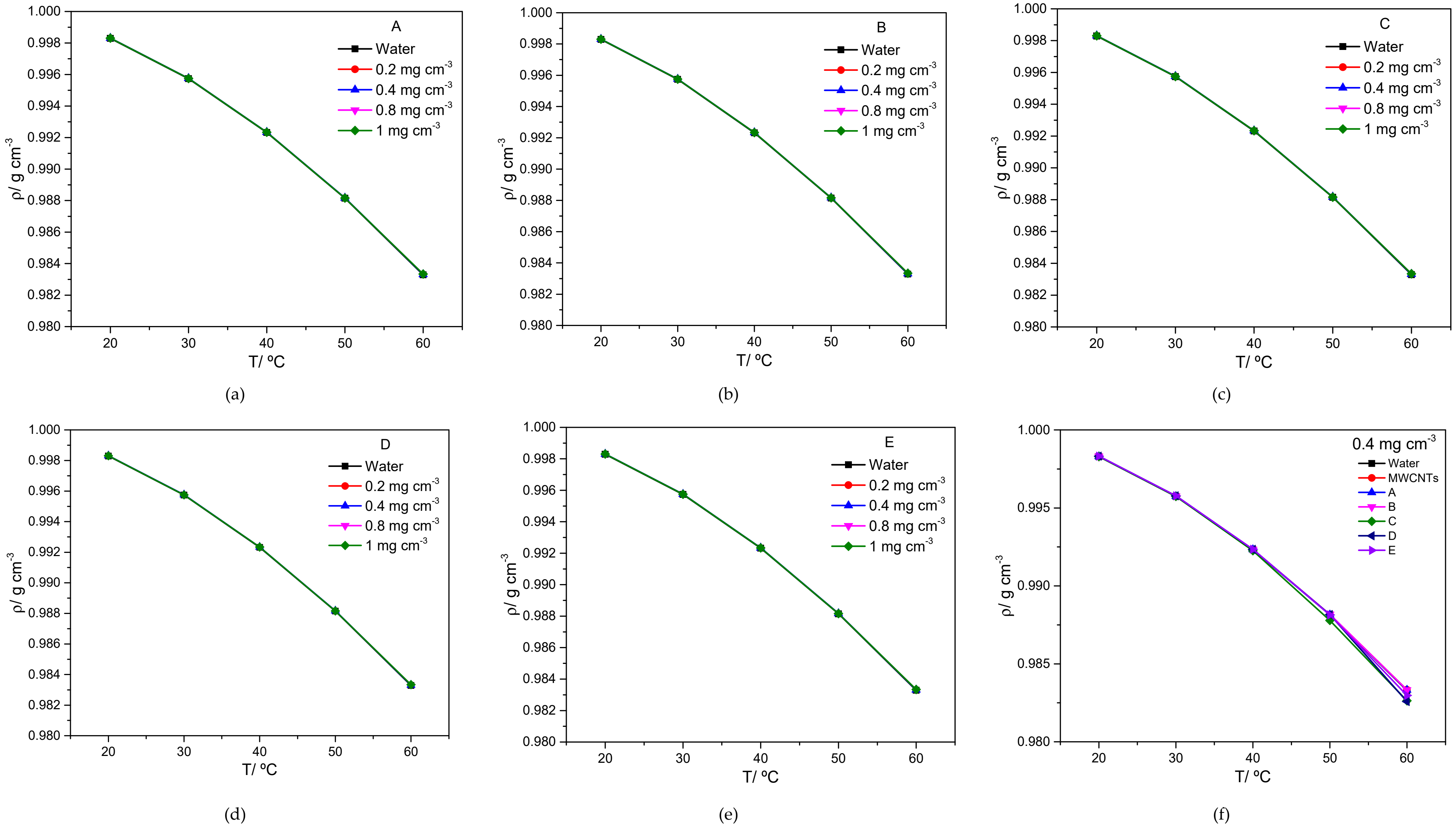

Figure S2. Densities (ρ/g cm^{-3}) of A – E mixtures (a-e) and comparison between samples at 0.4 mg cm^{-3} (f) in water as a function of temperature.

Table S4. Densities of MWCNTs and AgMWCNTs mixtures in water as a function of temperature for 0.4 mg cm^{-3}

	Water	MWCNTs	A	B	C	D	E
**Temperature/ °C**				**Density / g cm^{-3}**			
**60**	0.9833 ± 0.0012	0.9833 ± 0.0020	0.9833 ± 0.0033	0.9833 ± 0.0051	0.9826 ± 0.0034	0.9826 ± 0.0031	0.9830 ± 0.0004
**50**	0.9882 ± 0.0023	0.9882 ± 0.0031	0.9882 ± 0.0012	0.9882 ± 0.0023	0.9878 ± 0.0019	0.9882 ± 0.0054	0.9881 ± 0.0044
**40**	0.9923 ± 0.0009	0.9923 ± 0.0044	0.9923 ± 0.0019	0.9923 ± 0.0015	0.9923 ± 0.0066	0.9923 ± 0.0065	0.9924 ± 0.0055
**30**	0.9957 ± 0.0041	0.9958 ± 0.0032	0.9958 ± 0.0023	0.9958 ± 0.0033	0.9957 ± 0.0076	0.9958 ± 0.0076	0.9958 ± 0.0019
**20**	0.9983 ± 0.0024	0.9983 ± 0.0014	0.9983 ± 0.0066	0.9983 ± 0.0016	0.9983 ± 0.0009	0.9983 ± 0.0090	0.9983 ± 0.0043

Table S5. Densities of MWCNTs mixtures (0.2 – 1.0 mg cm^{-3}) in water as a function of temperature

	Water	0.2 mg cm^{-3}	0.4 mg cm^{-3}	0.8 mg cm^{-3}	1.0 mg cm^{-3}
**Temperature/ °C**			**Density / g cm^{-3}**		
**60**	0.9833 ± 0.0012	0.9833± 0.0011	0.9833 ± 0.0020	0.9834 ± 0.0044	0.9834 ± 0.0023
**50**	0.9882 ± 0.0023	0.9882± 0.0032	0.9882 ± 0.0031	0.9882 ± 0.0010	0.9882 ± 0.0095
**40**	0.9923 ± 0.0009	0.9923± 0.0022	0.9923 ± 0.0044	0.9924 ± 0.0022	0.9924 ± 0.0054
**30**	0.9957 ± 0.0041	0.9958± 0.0035	0.9958 ± 0.0032	0.9958 ± 0.0020	0.9958 ± 0.0033
**20**	0.9983 ± 0.0024	0.9983± 0.0009	0.9983 ± 0.0014	0.9983 ± 0.0054	0.9983 ± 0.0045

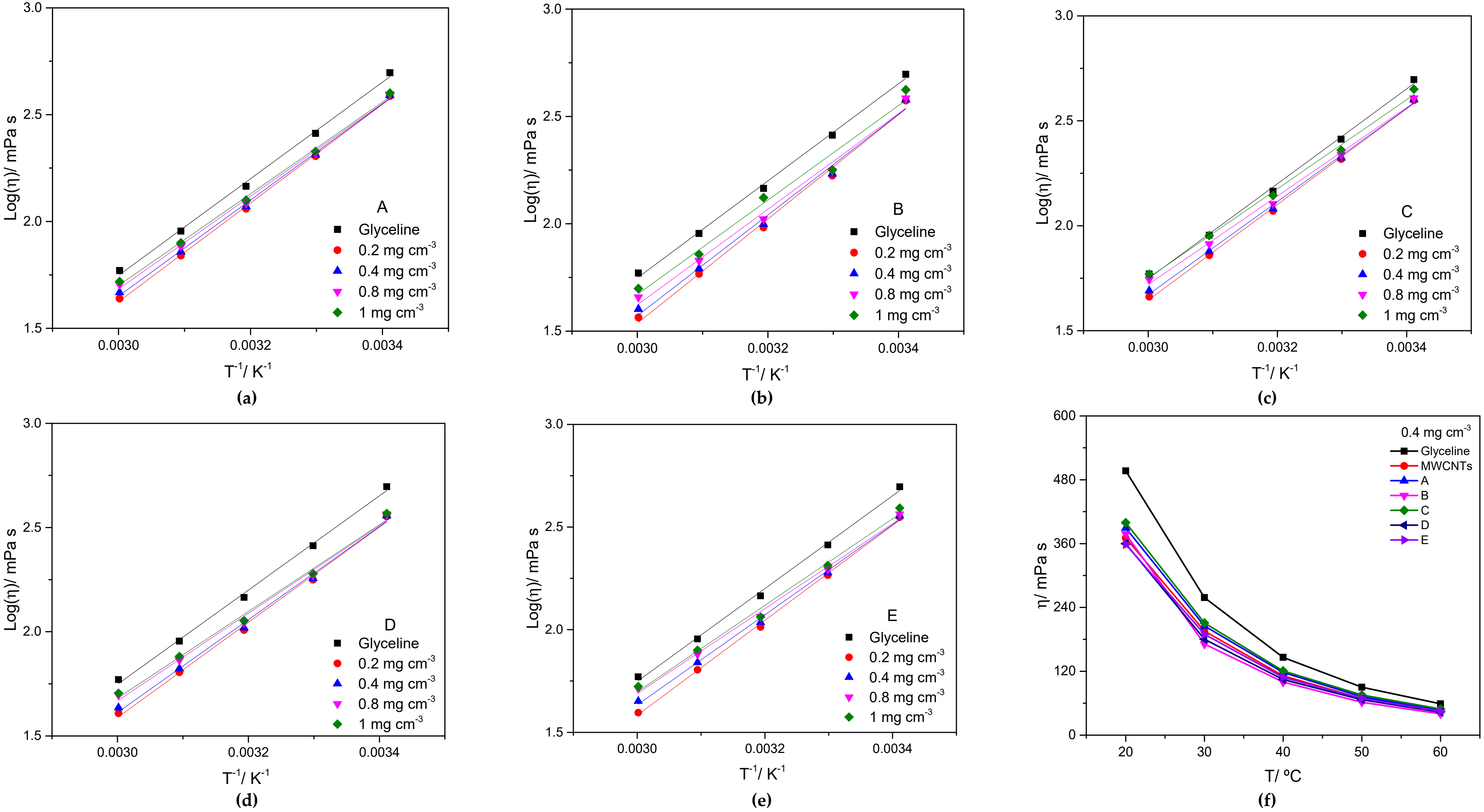

Figure S3. Plot of logarithm of the viscosity against the reciprocal value of the absolute temperature (1/T) for of A – E mixtures (a-e) and comparison between samples at 0.4 mg cm^{-3} (f) in glyceline.

Table S6. Viscosity of MWCNTs and AgMWCNTs mixtures in glyceline as a function of temperature for 0.4 mg cm^{-3}

	Glyceline	MWCNTs	A	B	C	D	E
**Temperature/ °C**	**Dynamic viscosity/ mPa s**						
**60**	58.93 ± 1.09	44.81 ± 2.44	46.58 ± 2.22	40.08 ± 4.32	49.05 ± 0.89	43.17 ± 4.11	44.88 ± 3.33
**50**	90.122 ± 1.55	68.99 ± 1.55	72.25 ± 4.12	61.85 ± 5.55	75.44 ± 1.43	66.48 ± 3.15	69.19 ± 1.55
**40**	146.13 ± 3.44	111.53 ± 4.32	117.64 ± 3.33	99.66 ± 3.21	120.73 ± 2.33	104.46 ± 4.01	108.56 ± 1.43
**30**	258.58 ± 6.89	195.56 ± 2.55	205.12 ± 3.89	171.06 ± 4.11	210.84 ± 4.31	180.24 ± 2.77	189.67 ± 3.33
**20**	496.8 ± 12.32	370.68 ± 3.54	389.35 ± 4.12	378.13 ± 2.88	399.38 ± 4.11	360.01 ± 4.33	358.48 ± 1.33

Table S7. Viscosity of MWCNTs mixtures (0.2 – 1.0 mg cm^{-3}) in glyceline as a function of temperature

	Glyceline	0.2 mg cm^{-3}	0.4 mg cm^{-3}	0.8 mg cm^{-3}	1.0 mg cm^{-3}
**Temperature/ °C**	**Dynamic viscosity/ mPa s**				
**60**	58.93 ± 1.09	42.81 ± 1.99	44.81 ± 2.44	51.69 ± 1.33	51.69 ± 3.12
**50**	90.122 ± 1.55	66.99 ± 1.54	68.99 ± 1.55	75.87 ± 1.50	75.87 ± 4.33
**40**	146.13 ± 3.44	109.53 ± 3.43	111.53 ± 4.32	118.41 ± 1.96	118.41 ± 1.59
**30**	258.58 ± 6.89	193.56 ± 8.98	195.56 ± 2.55	202.44 ± 1.33	202.44 ± 5.65
**20**	496.8 ± 12.32	368.68 ± 3.21	370.68 ± 3.54	377.56 ± 4.56	377.56 ± 8.12

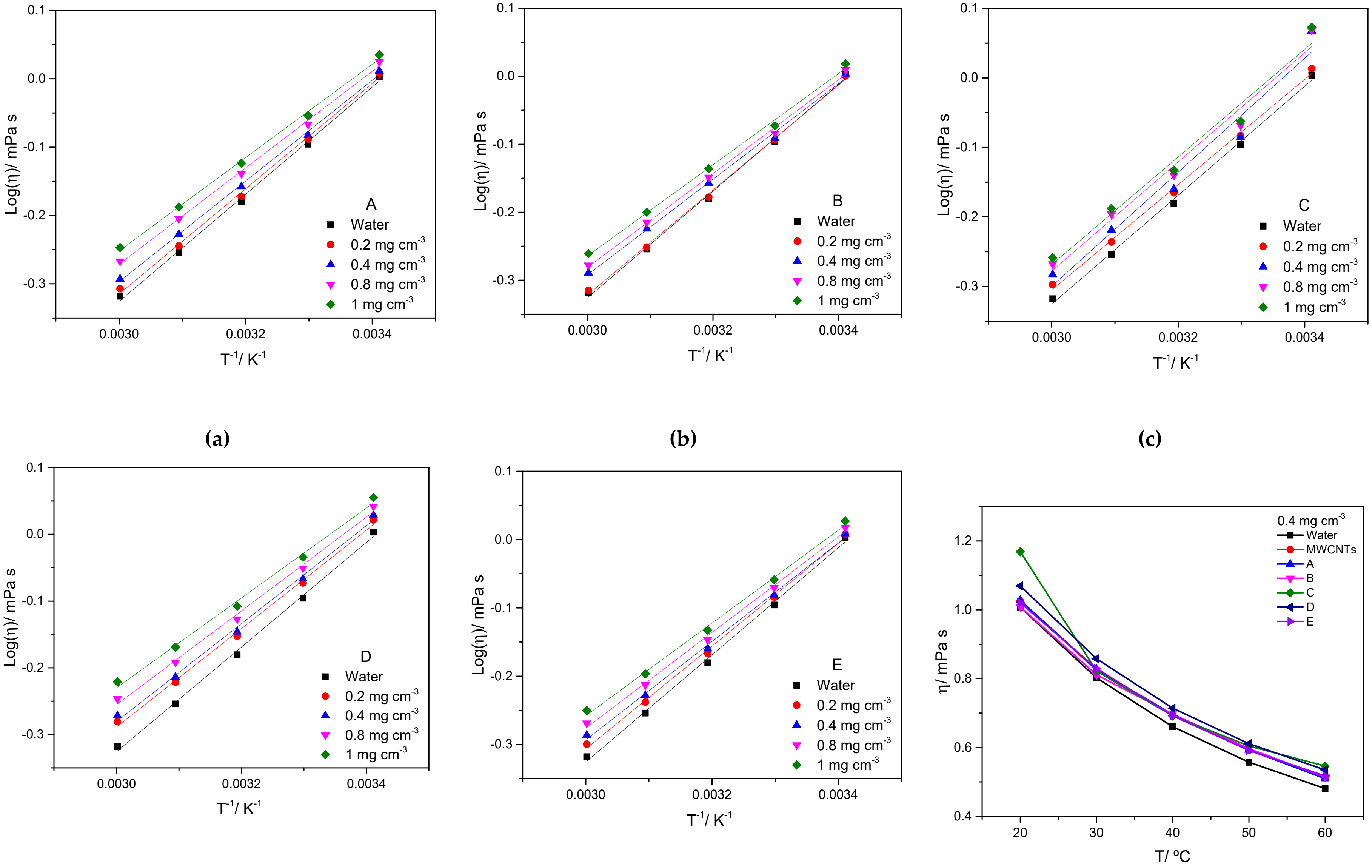

Log(η)/ mPa s
T-1/ K-1
A
B
C
D
E
Water
0.2 mg cm-3
0.4 mg cm-3
0.8 mg cm-3
1 mg cm-3
η/ mPa s
T/ ºC
0.4 mg cm-3
Water
MWCNTs
A
B
C
D
E

(a) **(b)** **(c)**

(d) **(e)** **(f)**

Figure S4. Plot of logarithm of the viscosity against the reciprocal value of the absolute temperature (1/T) for of A – E mixtures (a-e) and comparison between samples at 0.4 mg cm^{-3} (f) in water.

Table S8. Viscosity of MWCNTs and AgMWCNTs mixtures in water as a function of temperature for 0.4 mg cm^{-3}

	**Water**	**MWCNTs**	**A**	**B**	**C**	**D**	**E**
**Temperature/ °C**	**Dynamic viscosity/ mPa s**						
**60**	0.4807 ± 0.0312	0.5120 ± 0.0125	0.5096 ± 0.0231	0.5139 ± 0.0133	0.5460 ± 0.0065	0.5348 ± 0.0099	0.5174 ± 0.0431
**50**	0.5571 ± 0.0254	0.5919 ± 0.0543	0.5928 ± 0.0551	0.5965 ± 0.0341	0.6043 ± 0.0143	0.6112 ± 0.0154	0.5915 ± 0.0077
**40**	0.6604 ± 0.0356	0.6959 ± 0.0686	0.6958 ± 0.0321	0.6967 ± 0.0076	0.6918 ± 0.0043	0.7142 ± 0.0541	0.6922 ± 0.0312
**30**	0.8023 ± 0.0376	0.8246 ± 0.0361	0.8269 ± 0.0314	0.8111 ± 0.0031	0.8217 ± 0.0089	0.8577 ± 0.0654	0.8291 ± 0.0354
**20**	1.0074 ± 0.0576	1.0257 ± 0.0441	1.0272 ± 0.0412	1.0082 ± 0.0531	1.1685 ± 0.0067	1.0693 ± 0.0154	1.0203 ± 0.0546

Table S9. Viscosity of MWCNTs mixtures (0.2 – 1.0 mg cm^{-3}) in water as a function of temperature.

	**Water**	**0.2 mg cm^{-3}**	**0.4 mg cm^{-3}**	**0.8 mg cm^{-3}**	**1.0 mg cm^{-3}**
**Temperature/ °C**	**Dynamic viscosity/ mPa s**				
**60**	0.4807 ± 0.0312	0.5039 ± 0.0231	0.5120 ± 0.0125	0.5441 ± 0.0456	0.5962 ± 0.0433
**50**	0.5571 ± 0.0254	0.5803 ± 0.0311	0.5919 ± 0.0543	0.6240 ± 0.0777	0.6761 ± 0.0089
**40**	0.6604 ± 0.0356	0.6836 ± 0.0546	0.6959 ± 0.0686	0.7280 ± 0.0089	0.7801 ± 0.0087
**30**	0.8023 ± 0.0376	0.8255 ± 0.0125	0.8246 ± 0.0361	0.8567 ± 0.0656	0.9088 ± 0.0089
**20**	1.0074 ± 0.0576	1.0306 ± 0.0334	1.0257 ± 0.0441	1.0578 ± 0.0456	1.1099 ± 0.0008

Table S10. The calculated values of the activation energies of viscous flow (Eη) and conductivity (E$\boldsymbol{\tau}$) at different concentrations of MWCNTs and AgMWCNTs composites (A-E), in glyceline.

System		E_η/ kJ mol^{-1}	E_τ/ kJ mol^{-1}
Glyceline		18.79	17.55
MWCNTs	0.2 mg cm^{-3}	18.82	15.01
	0.4 mg cm^{-3}	18.61	13.61
	0.8 mg cm^{-3}	17.58	13.14
	1.0 mg cm^{-3}	16.94	9.92
A	0.2 mg cm^{-3}	18.78	11.92
	0.4 mg cm^{-3}	18.68	10.17
	0.8 mg cm^{-3}	18.04	9.09
	1.0 mg cm^{-3}	17.83	8.20
B	0.2 mg cm^{-3}	18.94	8.04
	0.4 mg cm^{-3}	18.65	7.29
	0.8 mg cm^{-3}	18.51	6.82
	1.0 mg cm^{-3}	18.28	6.28
C	0.2 mg cm^{-3}	18.12	11.23
	0.4 mg cm^{-3}	17.62	9.74
	0.8 mg cm^{-3}	17.52	9.10
	1.0 mg cm^{-3}	17.46	8.12
D	0.2 mg cm^{-3}	18.16	6.29
	0.4 mg cm^{-3}	17.94	5.54
	0.8 mg cm^{-3}	17.46	5.02
	1.0 mg cm^{-3}	17.28	4.78
E	0.2 mg cm^{-3}	18.39	3.77
	0.4 mg cm^{-3}	18.25	3.50
	0.8 mg cm^{-3}	18.19	3.32
	1.0 mg cm^{-3}	17.52	3.20

Table S11. The calculated values of the activation energies of viscous flow (Eη) and conductivity (E*τ*) at different concentrations of MWCNTs and AgMWCNTs composites (A-E), in water.

System		E_η/ kJ mol^{-1}	E_τ/ kJ mol^{-1}
Water		6.52	23.667
MWCNTs	0.2 mg cm^{-3}	6.30	17.25
	0.4 mg cm^{-3}	6.08	16.17
	0.8 mg cm^{-3}	5.82	15.96
	1.0 mg cm^{-3}	5.44	11.19
A	0.2 mg cm^{-3}	6.40	16.81
	0.4 mg cm^{-3}	6.13	17.22
	0.8 mg cm^{-3}	5.87	16.63
	1.0 mg cm^{-3}	5.67	10.57
B	0.2 mg cm^{-3}	6.41	12.44
	0.4 mg cm^{-3}	5.84	12.17
	0.8 mg cm^{-3}	5.73	10.99
	1.0 mg cm^{-3}	5.57	10.12
C	0.2 mg cm^{-3}	6.99	18.96
	0.4 mg cm^{-3}	6.82	16.167
	0.8 mg cm^{-3}	6.53	15.956
	1.0 mg cm^{-3}	6.44	11.19
D	0.2 mg cm^{-3}	6.13	17.20
	0.4 mg cm^{-3}	6.09	16.17
	0.8 mg cm^{-3}	5.84	15.96
	1.0 mg cm^{-3}	5.60	11.19
E	0.2 mg cm^{-3}	6.25	14.87
	0.4 mg cm^{-3}	5.99	14.76
	0.8 mg cm^{-3}	5.82	14.29
	1.0 mg cm^{-3}	5.64	12.54

Table S12. Viscosity–temperature model parameters for AgMWCNTs samples dispersed in glyceline.

Glyceline		AgMWCNTs concentration / mg cm^{-3}			
		**0.2**	**0.4**	**0.8**	**1.0**
**A**	**ln (η_0)**	-5.3 ± 0.1	-5.1 ± 0.2	-4.8 ± 0.4	-4.7 ± 0.3
	**E_η/R**	2309 ± 55	2248 ± 31	2170 ± 54	2145 ± 33
	**R^2**	0.997	0.998	0.999	0.996
**B**	**ln (η_0)**	-5.7 ± 0.2	-5.6 ± 0.4	-4.9 ± 0.4	-4.1 ± 0.3
	**E_η/R**	2427 ± 85	2358 ± 11	2151 ± 64	2115 ± 43
	**R^2**	0.994	0.998	0.991	0.995
**C**	**ln (η_0)**	-5.1 ± 0.1	-5.0 ± 0.1	-4.1 ± 0.4	-4.0 ± 0.1
	**E_η/R**	2311 ± 15	2245 ± 31	2150 ± 54	2131 ± 63
	**R^2**	0.994	0.991	0.919	0.976
**D**	**ln (η_0)**	-5.1 ± 0.1	-5.0 ± 0.4	-4.3 ± 0.4	-4.0 ± 0.1
	**E_η/R**	2312 ± 15	2249 ± 41	2182 ± 94	2131 ± 33
	**R^2**	0.999	0.998	0.998	0.991
**E**	**ln (η_0)**	-5.3 ± 0.1	-5.5 ± 0.1	-4.1 ± 0.2	-3.7 ± 0.3
	**E_η/R**	2349 ± 15	2278 ± 31	2271 ± 64	2245 ± 31
	**R^2**	0.999	0.991	0.999	0.996

Table S13. Viscosity–temperature model parameters for AgMWCNTs samples dispersed in water.

Water		AgMWCNTs concentration / mg cm^{-3}			
		**0.2**	**0.4**	**0.8**	**1.0**
	**ln (η_0)**	-2.62 ± 0.01	-2.51 ± 0.01	-2.39 ± 0.05	-2.29 ± 0.03
**A**	**E_η/R**	770 ± 31	737 ± 71	706 ± 51	682 ± 11
	**R^2**	0.999	0.998	0.991	0.997
	**ln (η_0)**	-2.63 ± 0.03	-2.41 ± 0.05	-2.35 ± 0.02	-2.19 ± 0.02
**B**	**E_η/R**	784 ± 21	703 ± 31	690 ± 51	677 ± 14
	**R^2**	0.997	0.999	0.991	0.997
	**ln (η_0)**	-2.53 ± 0.03	-2.41 ± 0.01	-2.29 ± 0.05	-2.19 ± 0.03
**C**	**E_η/R**	757 ± 30	713 ± 71	689 ± 31	612 ± 11
	**R^2**	0.999	0.996	0.992	0.998
	**ln (η_0)**	-2.59 ± 0.03	-2.47 ± 0.02	-2.19 ± 0.05	-2.09 ± 0.01
**D**	**E_η/R**	760 ± 38	727 ± 79	701 ± 49	667 ± 12
	**R^2**	0.991	0.998	0.992	0.998
	**ln (η_0)**	-2.69 ± 0.01	-2.57 ± 0.03	-2.29 ± 0.06	-2.00 ± 0.04
**E**	**E_η/R**	770 ± 58	717 ± 77	671 ± 44	601 ± 12
	**R^2**	0.994	0.999	0.999	0.988

Table S14. Surface tension of MWCNTs mixtures (0.2 – 1.0 mg cm^{-3}) in water as a function of temperature

	**Water**	**0.2 mg cm^{-3}**	**0.4 mg cm^{-3}**	**0.8 mg cm^{-3}**	**1.0 mg cm^{-3}**
**Temperature / °C**		**Surface tension/ mN.m^{-1}**			
**20**	72.00	71.11 ± 2.12	72.54 ± 1.11	74.76 ± 4.32	76.98 ± 0.32
**30**	71.71	70.897 ± 6.21	72.43 ± 4.31	74.53 ± 2.34	76.77 ± 2.43
**40**	71.44	70.68 ± 3.33	72.35 ± 8.12	74.29 ± 3.33	76.56 ± 4.44
**50**	70.68	70.67 ± 5.12	72.21 ± 1.34	74.01 ± 4.01	76.42 ± 2.98
**60**	70.33	70.47 ± 4.14	72.09 ± 5.44	73.77 ± 2.22	76.24 ± 5.44

Table S15. Surface tension of MWCNTs and AgMWCNTs mixtures in water as a function of temperature for 0.4 mg cm^{-3}

	**Water**	**MWCNTs**	**A**	**B**	**C**	**D**	**E**
**Temperature / °C**			**Surface tension / mN m^{-1}**				
**20**	--	72.54 ± 1.11	73.86 ± 2.43	74.09 ± 1.01	73.97 ± 0.98	74.29 ± 2.76	74.61± 4.31
**30**	71.71	72.43 ± 4.31	73.75 ± 2.01	73.98 ± 2.22	73.86± 1.56	74.19 ± 1.56	74.50 ± 5.45
**50**	71.44	72.35 ± 8.12	73.69 ± 2.32	73.90 ± 1.56	73.80 ± 1.11	74.11 ± 5.45	74.45 ± 3.21
**40**	70.68	72.21 ± 1.34	73.64 ± 2.45	73.87 ± 1.32	73.75 ± 3.24	74.07 ± 5.67	74.37 ± 5.67
**60**	70.33	72.09 ± 5.44	73.41 ± 3.33	73.64 ± 3.23	73.56 ± 1.89	73.84 ± 4.33	74.16 ± 3.12

Table S16. Surface tension of MWCNTs mixtures (0.2 – 1.0 mg cm^{-3}) in glyceline as a function of temperature.

	**Glyceline**	**0.2 mg cm^{-3}**	**0.4 mg cm^{-3}**	**0.8 mg cm^{-3}**	**1.0 mg cm^{-3}**
**Temperature/ °C**			**Surface tension / mN m^{-1}**		
**20**	56.33 ± 3.45	57.84 ± 1.43	59.27 ± 2.78	61.49 ± 2.12	63.71 ± 1.01
**30**	55.99 ± 3.43	57.497 ± 4.31	59.04 ± 3.11	61.17 ± 1.01	63.37 ± 2.22
**40**	54.11 ± 1.67	57.154 ± 1.21	58.81 ± 3.91	60.85 ± 2.89	63.02 ± 3.01
**50**	53.77 ± 1.09	56.897 ± 2.22	58.55 ± 3.01	60.57 ± 0.89	62.65 ± 1.78
**60**	53.31 ± 2.01	56.611 ± 0.98	58.37 ± 1.09	60.23 ± 1.09	62.28 ± 4.12

Table S17. Surface tension of MWCNTs and AgMWCNTs mixtures in glyceline as a function of temperature for 0.4 mg cm^{-3}.

	**Glyceline**	**MWCNTs**	**A**	**B**	**C**	**D**	**E**
**Temperature/ °C**				**Surface tension / mN m^{-1}**			
**20**	56.33 ± 3.45	59.27 ± 2.78	59.93 ± 1.66	60.59 ± 3.1	61.25 ± 4.12	61.91 ± 0.14	62.57 ± 1.23
**30**	55.99 ± 3.43	59.04 ± 3.11	59.698 ± 2.43	60.36 ± 1.81	61.02 ± 2.01	61.68 ± 1.33	62.34 ± 2.01
**50**	54.11 ± 1.67	58.81 ± 3.91	59.578 ± 1.08	60.23 ± 2.08	60.99 ± 1.78	61.55 ± 2.33	62.23 ± 1.09
**40**	53.77 ± 1.09	58.55 ± 3.01	59.466 ± 2.22	60.13 ± 3.33	60.79 ± 2.33	61.45 ± 2.67	62.11 ± 1.11
**60**	53.31 ± 2.01	58.37 ± 1.09	59.034 ± 3.31	59.69 ± 4.11	60.35 ± 3.01	61.01 ± 2.12	61.67 ± 2.71

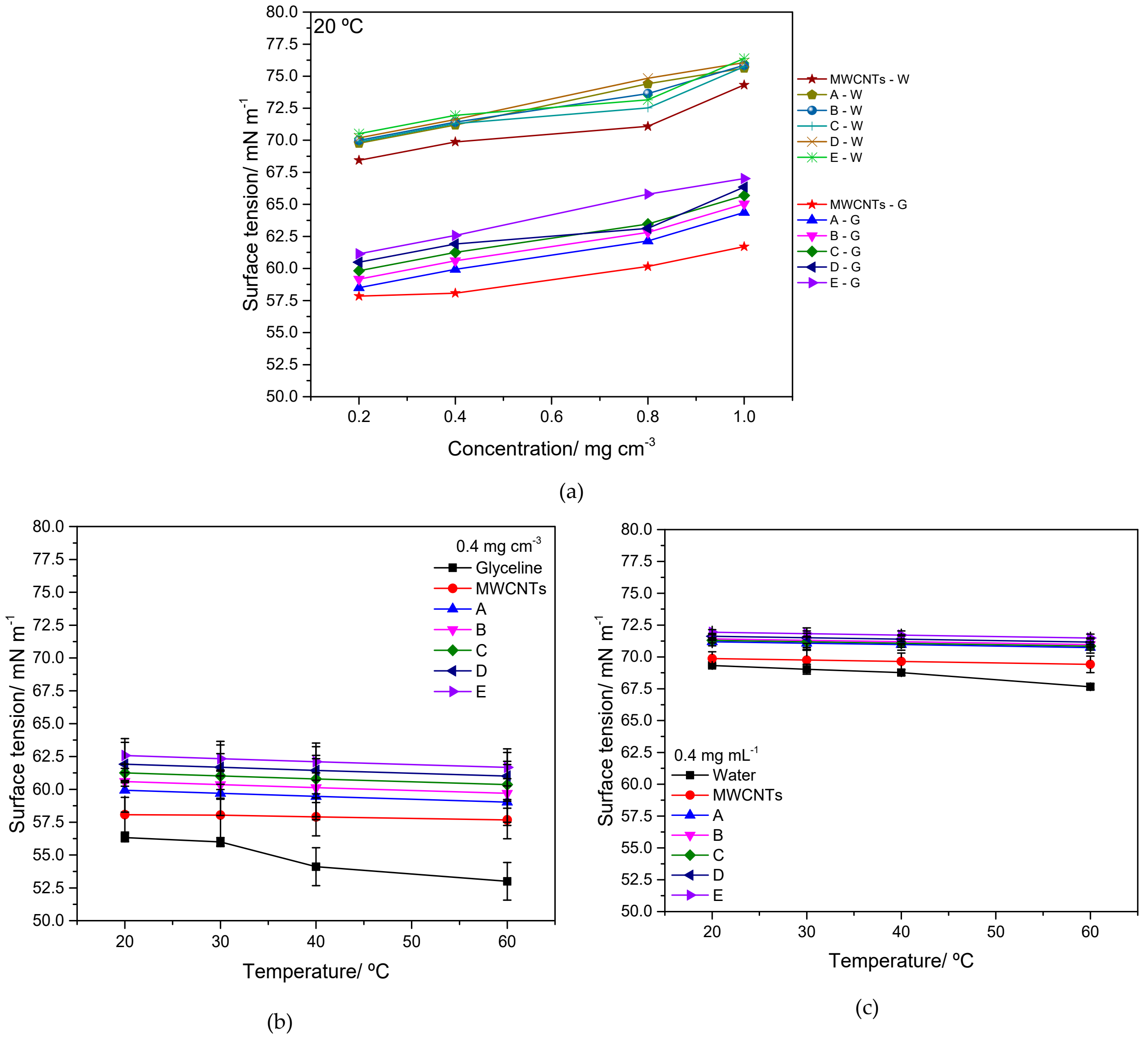

Figure S5. Surface tension of glyceline/ water mixtures with MWCNTs and AgMWCNTs as a function of carbon concentration (a) and temperature (b and c)

Table S18. Average hole size (Å) according to the Hole Theory for glyceline at different concentrations of AgMWCNTs composites (A-E) at 20 °C.

	$C/mg\ cm^{-3}$	A	B	C	D	E
Glyceline	0.2	4.389	4.365	4.341	4.317	4.294
	0.4	4.337	4.313	4.29	4.267	4.245
	0.8	4.259	4.236	4.214	4.226	4.139
	1	4.185	4.164	4.143	4.123	4.105

Table S19. Average hole size (Å) according to the Hole Theory for glyceline at different temperatures (20 °C – 60 °C) of AgMWCNTs composites (A-E) at 0.4 mg cm^{-3}

	Temperature/ K	A	B	C	D	E
Glyceline	293.15	4.337	4.313	4.29	4.267	4.245
	303.15	4.419	4.395	4.371	4.347	4.324
	313.15	4.502	4.475	4.451	4.427	4.403
	323.15	4.568	4.533	4.576	4.501	4.971
	333.15	4.658	4.633	4.607	4.582	4.557

Table S20. Conductivity of MWCNTs mixtures (0.2 – 1.0 mg cm^{-3}) in water as a function of temperature

	**Water**	**0.2 mg cm^{-3}**	**0.4 mg cm^{-3}**	**0.8 mg cm^{-3}**	**1.0 mg cm^{-3}**
**Temperature/ °C**	**Ionic conductivity/ mS cm^{-1}**				
**20**	0.050 ± 0.004	0.124 ± 0.009	0.154 ± 0.008	0.241 ± 0.015	0.472 ± 0.006
**30**	0.055 ± 0.001	0.674 ± 0.010	0.712 ± 0.012	0.793 ± 0.021	1.022 ± 0.043
**40**	0.061 ± 0.010	1.463 ± 0.014	1.489 ± 0.009	1.579 ± 0.007	1.809 ± 0.055
**50**	0.067 ± 0.006	2.444 ± 0.008	2.545 ± 0.013	2.777 ± 0.031	2.999 ± 0.024
**60**	0.071 ± 0.008	3.252 ± 0.021	3.278 ± 0.007	3.368 ± 0.043	3.598 ± 0.066

Table S21. Conductivity of MWCNTs and AgMWCNTs mixtures in water as a function of temperature for 0.4 mg cm^{-3}

	**Water**	**MWCNTs**	**A**	**B**	**C**	**D**	**E**
**Temperature/ °C**	**Ionic conductivity/ mS cm^{-1}**						
**20**	0.050 ± 0.004	0.154 ± 0.008	0.251 ± 0.005	0.409 ± 0.006	0.376 ± 0.043	0.555 ± 0.043	0.712 ± 0.087
**30**	0.055 ± 0.001	0.712 ± 0.012	0.832 ± 0.004	0.951 ± 0.011	0.926 ± 0.006	1.105 ± 0.033	1.261 ± 0.099
**40**	0.061 ± 0.010	1.489 ± 0.009	1.589 ± 0.014	1.739 ± 0.056	1.715 ± 0.033	1.894 ± 0.067	2.049 ± 0.101
**50**	0.067 ± 0.006	2.545 ± 0.013	2.768 ± 0.021	2.987 ± 0.088	1.876 ± 0.015	3.121 ± 0.004	3.771 ± 0.069
**60**	0.071 ± 0.008	3.278 ± 0.007	3.378 ± 0.033	3.528 ± 0.055	3.504 ± 0.076	3.622 ± 0.089	3.838 ± 0.046

Table S22. Conductivity of MWCNTs mixtures (0.2 – 1.0 mg cm^{-3}) in glyceline as a function of temperature.

	Glyceline	0.2 mg cm^{-3}	0.4 mg cm^{-3}	0.8 mg cm^{-3}	1.0 mg cm^{-3}
**Temperature/ °C**	**Ionic conductivity/ mS cm^{-1}**				
**20**	0.407 ± 0.013	0.897 ± 0.003	2.023 ± 0.143	2.741 ± 0.431	4.061 ± 0.065
**30**	0.913 ± 0.045	4.217 ± 0.043	5.343 ± 0.231	6.061 ± 0.198	7.381 ± 0.156
**40**	1.601 ± 0.032	6.517 ± 0.076	7.643 ± 0.190	8.361 ± 0.089	9.681 ± 0.787
**50**	2.888 ± 0.012	10.432 ± 0.081	11.555 ± 0.512	12.656 ± 0.561	13.765 ± 0.456
**60**	3.099 ± 0.112	11.847 ± 0.056	12.973 ± 0.897	13.691 ± 0.546	15.011 ± 0.891

Table S23. Conductivity of MWCNTs and AgMWCNTs mixtures in glyceline as a function of temperature for 0.4 mg cm^{-3}.

	Glyceline	MWCNTs	A	B	C	D	E
**Temperature/ °C**	**Ionic conductivity/ mS cm^{-1}**						
**20**	0.407 ± 0.013	2.023 ± 0.143	4.963 ± 0.678	8.692 ± 0.432	5.542 ± 0.154	13.036 ± 0.234	23.242 ± 0.456
**30**	0.913 ± 0.045	5.343 ± 0.231	6.283 ± 0.586	10.012 ± 0.333	6.862 ± 0.796	14.356 ± 0.155	24.562 ± 0.624
**40**	1.601 ± 0.032	7.643 ± 0.190	10.583 ± 0.876	14.312 ± 0.678	11.162 ± 0.444	18.656 ± 0.116	27.862 ± 0.326
**50**	2.888 ± 0.012	11.555 ± 0.512	11.213 ± 0.945	15.898 ± 0.089	12.453 ± 0.154	20.981 ± 0.178	31.123 ± 0.141
**60**	3.099 ± 0.112	12.973 ± 0.897	15.913 ± 0.453	19.642 ± 0.768	16.492 ± 0.313	23.986 ± 0.434	34.192 ± 0.928

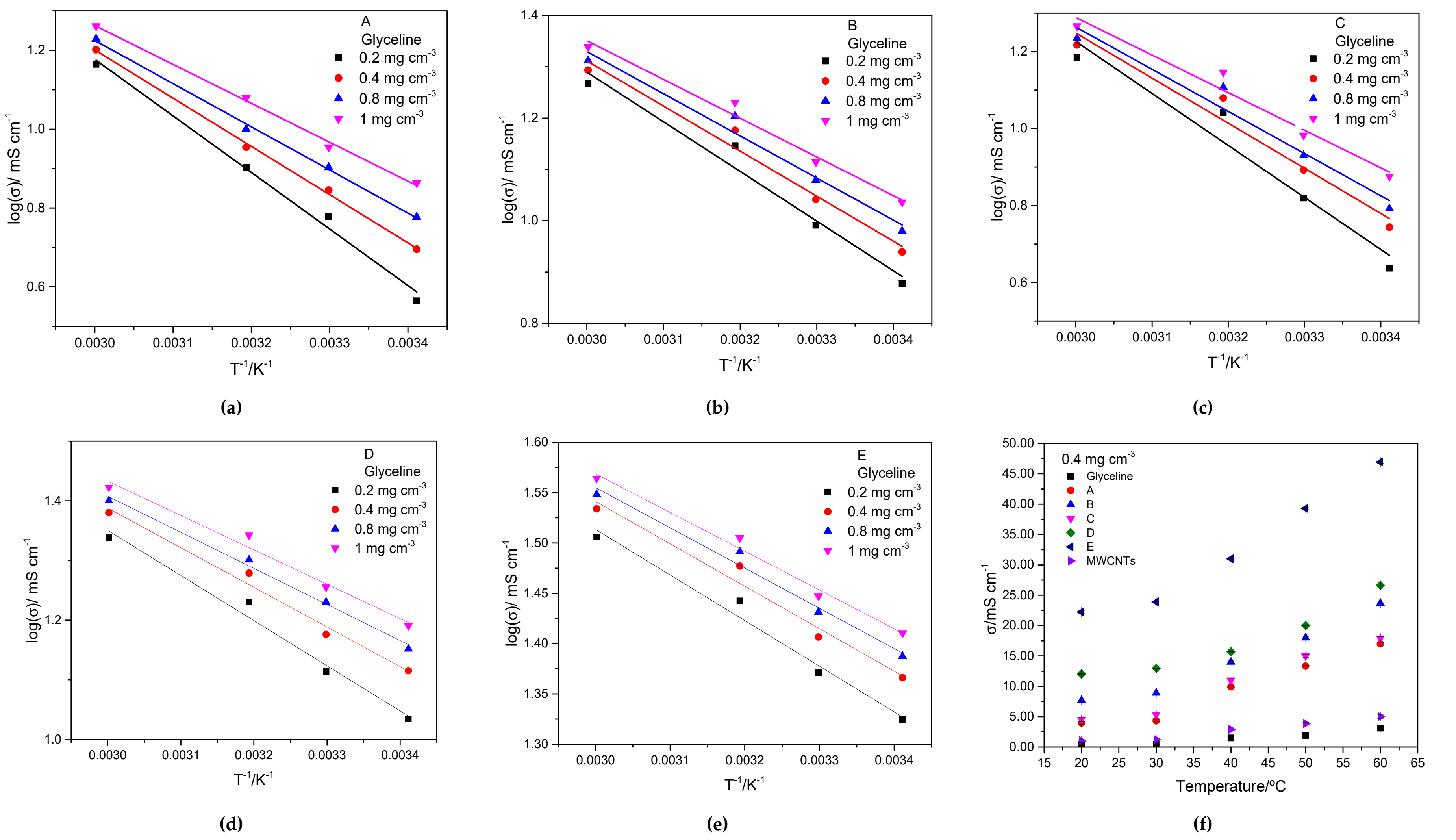

Figure S6. Plot of logarithm of the conductivity against the reciprocal value of the absolute temperature (1/T) for of A – E mixtures (a-e) and comparison between samples at 0.4 mg cm^{-3} (f) in glyceline.

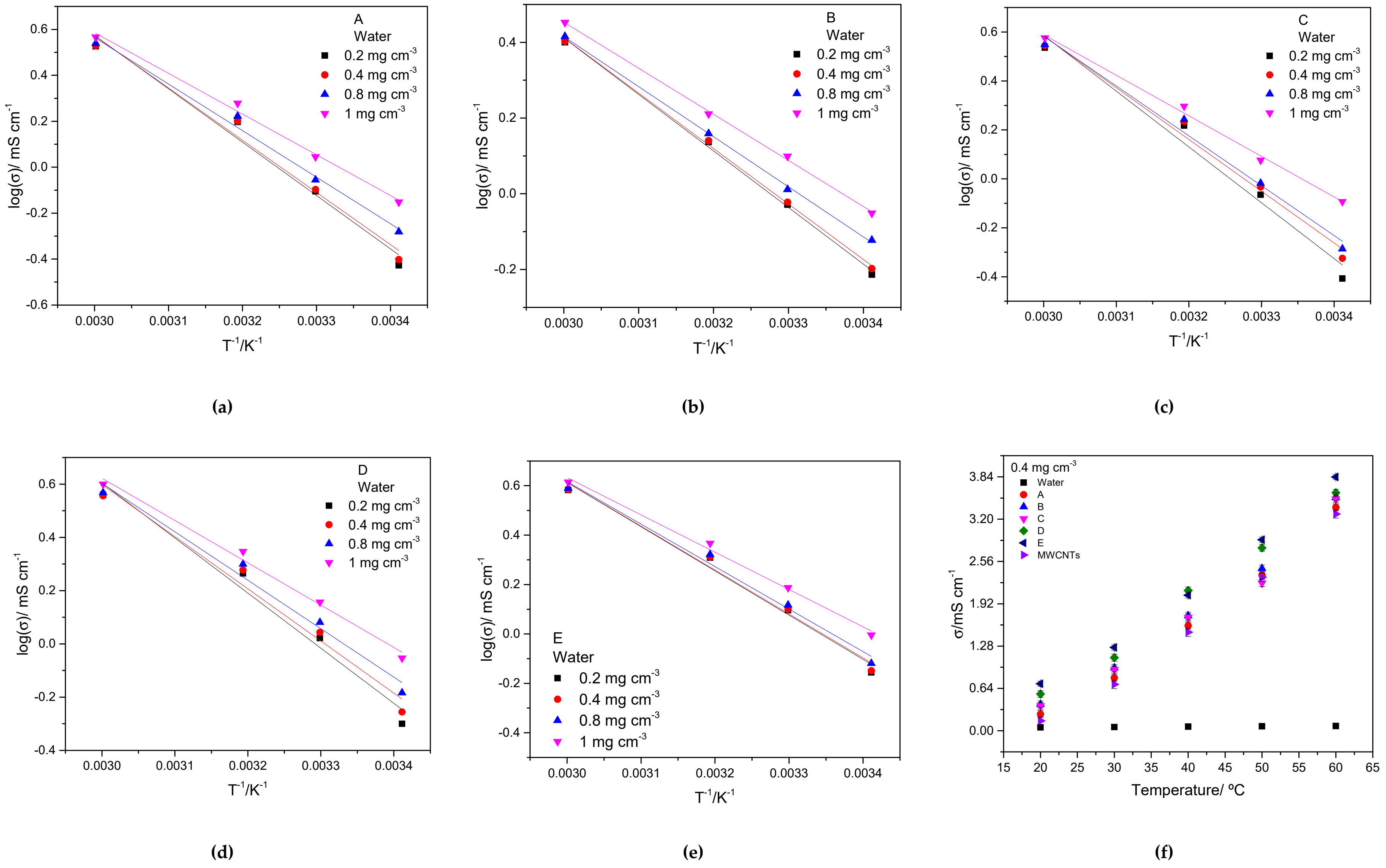

Figure S7. Plot of logarithm of the conductivity against the reciprocal value of the absolute temperature (1/T) for of A – E mixtures (a-e) and comparison between samples at 0.4 mg cm^{-3} (f) in water.

Table S24. Conductivity–temperature model parameters for AgMWCNTs samples dispersed in glyceline.

Glyceline		AgMWCNTs concentration / mg cm^{-3}			
		**0.2**	**0.4**	**0.8**	**1.0**
	**ln (σ_0)**	5.48 ± 0.08	4.87 ± 0.11	4.51 ± 0.06	4.22 ± 0.09
**A**	**E_σ/R**	-1434 ± 77	-1233 ± 88	-1094 ± 91	-987 ± 55
	**R^2**	0.997	0.991	0.999	0.996
	**ln (σ_0)**	5.28 ± 0.11	4.51 ± 0.21	4.00 ± 0.01	3.18 ± 0.19
**B**	**E_σ/R**	-1404 ± 97	-1313 ± 108	-1064 ± 92	-917 ± 95
	**R^2**	0.999	0.999	0.999	0.998
	**ln (σ_0)**	5.38 ± 0.01	4.81 ± 0.31	4.01 ± 0.22	4.05 ± 0.22
**C**	**E_σ/R**	-1334 ± 78	-1223 ± 28	-1004 ± 88	-907 ± 88
	**R^2**	0.999	0.991	0.990	0.999
	**ln (σ_0)**	5.21 ± 0.08	4.33 ± 0.01	4.01 ± 0.26	4.02 ± 0.09
**D**	**E_σ/R**	-1211 ± 99	-1133 ± 18	-1001 ± 99	-911 ± 15
	**R^2**	0.999	0.990	0.991	0.996
	**ln (σ_0)**	5.48 ± 0.08	4.87 ± 0.11	4.51 ± 0.06	4.22 ± 0.09
**E**	**E_σ/R**	-1434 ± 77	-1233 ± 88	-1094 ± 91	-987 ± 55
	**R^2**	0.997	0.991	0.999	0.996

Table S25. Conductivity–temperature model parameters for AgMWCNTs samples dispersed in water.

Water		AgMWCNTs concentration / mg cm^{-3}			
		**0.2**	**0.4**	**0.8**	**1.0**
	**ln (σ_0)**	7.54 ± 0.13	7.38 ± 0.22	6.64 ± 0.33	5.90 ± 0.51
**A**	**E_σ/R**	-2323 ± 101	-2272 ± 88	-2026 ± 100	-1772 ± 105
	**R^2**	0.991	0.997	0.999	0.995
	**ln (σ_0)**	7.04 ± 0.22	6.88 ± 0.52	6.14 ± 0.13	5.80 ± 0.99
**B**	**E_σ/R**	-2221 ± 102	-2132 ± 108	-2006 ± 90	-1742 ± 115
	**R^2**	0.999	0.999	0.991	0.995
	**ln (σ_0)**	7.11 ± 0.13	7.58 ± 0.12	6.84 ± 0.31	5.97 ± 0.41
**C**	**E_σ/R**	-2313 ± 101	-2244 ± 118	-2126 ± 90	-2002 ± 15
	**R^2**	0.997	0.999	0.999	0.995
	**ln (σ_0)**	7.66 ± 0.23	7.18 ± 0.55	6.04 ± 0.31	5.91 ± 0.41
**D**	**E_σ/R**	-2300 ± 11	-2172 ± 68	-2000 ± 99	-1692 ± 15
	**R^2**	0.993	0.994	0.999	0.995
	**ln (σ_0)**	5.98 ± 0.10	7.94 ± 0.22	5.77 ± 0.33	5.10 ± 0.51
**E**	**E_σ/R**	-1788 ± 91	-1662 ± 88	-1526 ± 10	-1472 ± 105
	**R^2**	0.991	0.997	0.999	0.995

Table S26. Refractive index of MWCNTs mixtures (0.2 – 1.0 mg cm^{-3}) in water as a function of temperature

Temperature / °C	0.2 mg cm^{-3}	0.4 mg cm^{-3}	0.8 mg cm^{-3}	1.0 mg cm^{-3}
**20**	1.33695 ± 0.00021	1.34095 ± 0.00030	1.35325 ± 0.00094	1.38655 ± 0.00011
**30**	1.32545 ± 0.00012	1.33241 ± 0.00077	1.35012 ± 0.00032	1.38111 ± 0.00034
**40**	1.32012 ± 0.00033	1.33013 ± 0.00031	1.34765 ± 0.00067	1.37678 ± 0.00021
**50**	1.31131 ± 0.00001	1.323561 ± 0.00076	1.34341 ± 0.00056	1.37012 ± 0.00078
**60**	1.30111 ± 0.00055	1.32014 ± 0.00088	1.33871 ± 0.00068	1.36765 ± 0.00044

Table S27. Refractive index of MWCNTs and AgMWCNTs mixtures in water as a function of concentration at 20 °C.

	MWCNTs	A	B	C	D	E
**0.2**	1.33695 ± 0.00001	1.34445 ± 0.00021	1.34531 ± 0.00032	1.34425 ± 0.00043	1.34426 ± 0.00041	1.34695 ± 0.00065
**0.4**	1.34095 ± 0.00030	1.34845 ± 0.00043	1.34954 ± 0.00014	1.34825 ± 0.00036	1.34826 ± 0.00008	1.35095 ± 0.00043
**0.8**	1.35325 ± 0.00023	1.36075 ± 0.00026	1.36181 ± 0.00054	1.36055 ± 0.00067	1.36057 ± 0.00089	1.36325 ± 0.00076
**1**	1.38655 ± 0.00055	1.39405 ± 0.00099	1.39510 ± 0.00087	1.39385 ± 0.00083	1.39386 ± 0.00043	1.39655 ± 0.00035

Table S28. Refractive index of MWCNTs mixtures (0.2 – 1.0 mg cm^{-3}) in glyceline as a function of temperature

Temperature/ °C	Glyceline	0.2 mg. cm^{-3}	0.4 mg. cm^{-3}	0.8 mg. cm^{-3}	1.0 mg. cm^{-3}
**20**	1.33311 ± 0.00032	1.48764 ± 0.00032	1.49065 ± 0.00065	1.51183 ± 0.00014	1.55321 ± 0.00043
**30**	1.32315 ± 0.00013	1.47655 ± 0.00055	1.48654 ± 0.00043	1.50652 ± 0.00054	1.54652 ± 0.00034
**40**	1.32010 ± 0.00023	1.45654± 0.00065	1.47653 ± 0.00034	1.49654 ± 0.00034	1.53654 ± 0.00065
**50**	1.31318 ± 0.00076	1.45087 ± 0.00035	1.47087 ± 0.00033	1.48709 ± 0.00088	1.52709 ± 0.00077
**60**	1.30993 ± 0.00044	1.44763 ± 0.00076	1.46764 ± 0.00067	1.47765± 0.00078	1.51763 ± 0.00101

Table S29. Refractive index of MWCNTs and AgMWCNTs mixtures in glyceline as a function of concentration at 20 °C

[C]/ mg cm^{-3}	MWCNTs	A	B	C	D	E
**0.2**	1.48735 ± 0.00033	1.50251 ± 0.00023	1.50131 ± 0.00076	1.50645 ± 0.00059	1.50429 ± 0.00043	1.49755 ± 0.00089
**0.4**	1.49065 ± 0.00065	1.50550 ± 0.00038	1.49910 ± 0.00041	1.50945 ± 0.00043	1.50723 ± 0.00009	1.49955 ± 0.00004
**0.8**	1.51155 ± 0.00012	1.52673 ± 0.00028	1.52431 ± 0.00084	1.53065 ± 0.00012	1.52844 ± 0.00065	1.52755 ± 0.00041
**1**	1.54275 ± 0.00034	1.56798 ± 0.00087	1.57098 ± 0.00099	1.57185 ± 0.00071	1.56963 ± 0.00067	1.55755 ± 0.00067

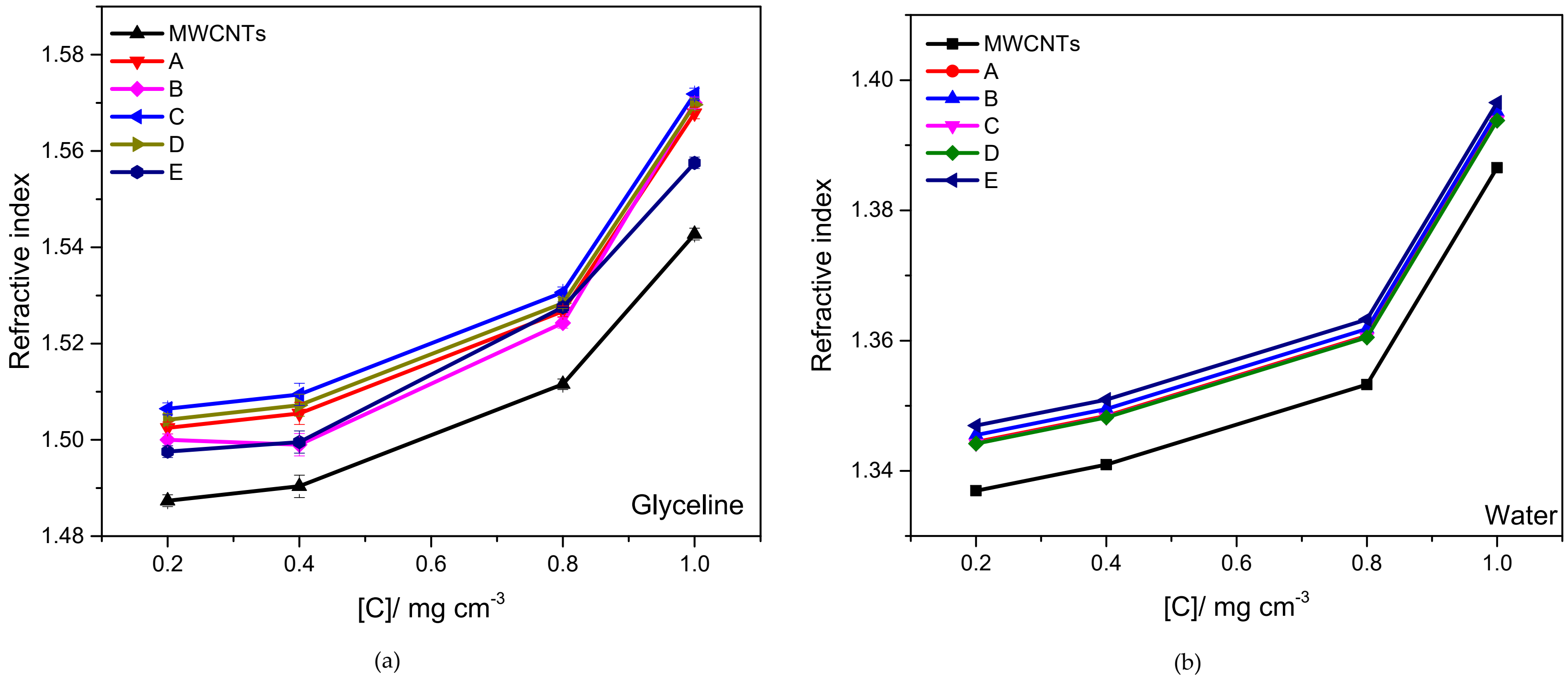

Figure S8. Effect of concentration of MWCNTs and AgMWCNTs composites on refractive index in glyceline (a) and water (b) media, at 20 °C

Statistical analysis

Table S30. Two-way ANOVA between the groups of MWCNTs and AgMWCNTs samples dispersed in glyceline with different temperatures (20 °C – 60 °C) and concentrations (0 – 1.0 mg cm^{-3}).

Dispersion media	Properties	Source of variation	Sum of squares	Degree of freedom	Mean square	F	P value
Glyceline	Viscosity	Interaction	1487	20	74.37	0.0035	> 0.9999
		Concentration	60437	4	15109	0.7311	0.0325
		Temperature	5683	5	1137	0.0550	0.0480
	Density	Interaction	9.004×10^{-6}	20	4.502×10^{-7}	0.0059	>0.9999
		Concentration	2.825×10^{-6}	4	7.063×10^{-7}	0.0093	0.9998
		Temperature	1.510×10^{-5}	5	3.020×10^{-6}	0.0396	0.0391
	Conductivity	Interaction	1183	20	59.16	3.047	<0.0001
		Concentration	4970	4	1242	63.99	<0.0001
		Temperature	4695	5	938.9	48.36	<0.0001
	Surface Tension	Interaction	47.73	20	2.386	1.992	0.0121
		Concentration	1617	4	404.4	337.6	<0.0001
		Temperature	139.8	5	27.95	23.34	<0.0001
	RI	Interaction	112.3	20	12.43	12.43	0.0785
		Concentration	12341	4	102.32	11.67	0.9991
		Temperature	11.43	5	90.89	9.09	0.9951

Table S31. Two-way ANOVA between the groups of MWCNTs and AgMWCNTs samples dispersed in water with different temperatures (20 °C – 60 °C) and concentrations (0 – 1.0 mg cm^{-3}).

Dispersion media	Properties	Source of variation	Sum of squares	Degree of freedom	Mean square	F	P-value
Water	Viscosity	Interaction	0.0134	20	0.0007	0.0154	>0.9999
		Concentration	0.1586	4	0.0396	0.9084	0.4615
		Temperature	0.0252	5	0.0051	0.1157	0.9887
	Density	Interaction	1.124×10^{-7}	20	5.622×10^{-9}	0.00015	>0.9999
		Concentration	1.742×10^{-8}	4	4.354×10^{-9}	0.00012	>0.9999
		Temperature	1.745×10^{-7}	5	3.491×10^{-8}	0.00096	>0.9999
	Conductivity	Interaction	0.2337	20	0.0155	0.0094	>0.9999
		Concentration	2.130	4	0.7101	0.4267	0.7343
		Temperature	4.834	5	0.9669	0.5810	0.7145
	Surface Tension	Interaction	10.22	20	0.5110	3.176	<0.0001
		Concentration	884.5	4	221.1	1374	<0.0001
		Temperature	40.81	5	8.161	50.72	<0.0001
	RI	Interaction	13.45	20	1.453×10^{-6}	11.45	>0.9999
		Concentration	389.1	4	3.122×10^{-9}	0.0123	>0.9999
		Temperature	31.9	5	5.465×10^{-5}	12.45	>0.9999
